# Cancer cells metastasize in several types of cancer via two distinct routes, probably based on mechanical signals

**DOI:** 10.3389/fcell.2026.1755547

**Published:** 2026-04-30

**Authors:** Claudia Tanja Mierke

**Affiliations:** Retired, Werdau, Germany

**Keywords:** angiotropism, intravascular cancer cell dissemination, mechanosensing, mechanotransduction, pericyte mimicry, stiffness, tumor microenvironment, vascular mimicry

## Abstract

Tumor diseases pose an enormous health threat when they become malignant, as in the process of metastasis. Cancer metastasis has been extensively studied in the past, but attention has been focused primarily on the established intraluminal metastasis route. Research efforts have concentrated on the complex mechanisms behind this process based on biochemical and molecular analyses. Therefore, a comprehensive knowledge of the mechanobiological principles of cancer metastasis and the identification of key molecules and signaling pathways involving mechanical cues are of the utmost importance. Moreover, there are still significant knowledge gaps regarding the two fundamentally different major routes of metastasis, encompassing key mechanosignaling pathways, altered regulation of organ-specific tropism mechanisms, and the impact of the microenvironment, such as mechanical cues. This review examines the stepwise events of cancer metastasis by pointing out the two distinct major routes: intraluminal and extraluminal metastasis. It clarifies the specific processes that underlie the two major metastasis routes with emphasis on mechanobiological characteristics. In addition to classical biochemical features, the review emphasizes the impact of the tumor microenvironment, which is understood to have an immunosuppressive role and seems to mechanically stimulate cancer cells, causing changes in their functional characteristics. The review explores whether the extraluminal metastasis (angiotropism) route of cancer cells could help them to evade mechanical forces that arise during extravasation and intravasation and circumvent the unfavorable environment in hematopoietic or lymphatic vessels, such as forces generated within the vessel lumen caused by the blood flow. The overview explains the advantages and disadvantages of the two major metastasis pathways. It identifies the molecules and signal transduction pathways involved in each pathway. Finally, it is discussed whether these two routes—and possibly a new third interfacial metastasis route—are generally interchangeable and universal in cancer metastasis rather than limited to solely specific cancer types. In addition, the possibility that switching between metastasis pathways serves as an additional regulatory mechanism for cancer metastasis is evaluated. Moreover, the interaction of cancer cells with immune cells, such as neutrophils, is highlighted in both metastasis pathways. Ultimately, it is argued that a more detailed analysis of the relatively new alternative metastasis routes (namely, the extracellular and interfacial metastasis routes) could optimize future treatment options for metastatic cancer.

## 1 Introduction

The most deadly facet of cancer is certainly metastasis, whereby the formation of secondary tumors is the cause of the majority of cancer-related mortalities (Steeg, 2016). The cancer progression that ultimately develops into these deadly secondary tumors proceeds through a metastatic cascade and encompasses various consecutive steps. In short, cancer begins when a healthy cell undergoes mutations and epigenetic alterations that result in out-of-control proliferation and growth of an initial primary solid tumor within healthy tissue (Grandér, 1998; Vicente-Dueñas et al., 2018). Initial tumor growth is concomitant with the establishment of a beneficial microenvironment (Werb and Lu, 2015), particularly due to angiogenesis/lymphangiogenesis in the vicinity of the primary solid tumor via newly generated blood/lymphatic vessels (Watnick, 2012; Stacker et al., 2014), an inflammation-like immune reaction (Whiteside, 2006), and a reshaping of the ambient extracellular matrix (ECM) (Alexander and Cukierman, 2016). Simultaneously, certain cancer cells of the primary tumor gain new features that enable them to leave the primary solid tumor and infiltrate the nearby stroma or travel alongside adjacent blood vessels (Gritsenko et al., 2017). This is when the process of cancer metastasis begins.

In cancer, the capability to form metastases, which cause the disease to progress malignantly, plays a decisive role in patient survival and the choice of optimal treatment options (Ganesh and Massagué, 2021). In the past, it was always assumed that the mechanisms contributing to cancer metastasis were essentially universal. The concept of the universality of cancer metastasis is widely accepted worldwide, particularly in the field of tumor mechanobiology and tumor microenvironment (TME) research, where it remains the most prevalent concept. It is now becoming apparent that the metastasis process via the intraluminal route is not the same for all tumors. This is most evident in certain types of tumors, such as melanoma and glioma, in which an extraluminal route is exploited for cancer metastasis. The concept of universality is increasingly the subject of controversial debate (Mierke, 2025c). Nevertheless, due to tumor-specific differences in cancer, such as the specific selection of target organs for cancer metastasis, it remains very difficult to fully comprehend the process of metastasis. It is even more difficult to effectively prevent the metastasis process with pharmaceuticals, especially in view of the role of mechanical influences in the metastasis process. It is currently still difficult to predict whether a primary tumor will progress to a malignant tumor through the development of metastases. There is an ongoing demand for novel markers, such as physical markers, to identify disseminated cancer cells and their specific malignant potential. The mechanobiological principles of cancer metastasis play a decisive role, since the process of cancer metastasis, i.e., the dissemination of cancer cells from a primary tumor to specific distant target areas, is strongly determined by these principles. There are principles governing how cancer cells actually engage with their physical environment and how these interactions influence cell performance throughout the metastasis cascade. Mechanobiological principles are usually directly related to the environment of the primary tumor and therefore cannot be considered independently of it, which makes them highly relevant. Not only the interaction of the cancer cells with their environment but also the interaction of the TME with the cancer cells play crucial roles in the process of metastasis (Mierke, 2019; 2020; 2021a). In the past, integrins have been extensively studied as bidirectional connections linking the ECM and the cytoskeleton of cells (Geiger et al., 2001; Mierke et al., 2011; Mierke, 2013a; Elosegui-Artola et al., 2014) and between endothelial cells and cancer cells during the intraluminal metastatic journey. In addition, the sensing of the stiffness can be performed through integrins (Elosegui-Artola et al., 2014) that can impact the choice of the migration and invasion mode. The extraluminal pathway of cancer metastasis, referred to as angiotropism or extravascular migratory metastasis (EVMM) (see Table 1 for definitions), is less well understood, but appears to be an alternative metastatic pathway not only for melanoma (Lugassy et al., 1998; Moy et al., 2019) and melanocytic tumors (Barnhill et al., 2011; Wilsher et al., 2013) but also for a growing number of other cancers, such as breast cancer (Çalım Gürbüz et al., 2024), glioma (Lugassy et al., 2002), leukemia, pancreatic cancer (Levy et al., 2009), and prostatic cancer (Lugassy et al., 2005).

**TABLE 1:** Important definitions.

Specific terms	Definitions
Angiotropism	It represents to a cancer mechanism in which malignant cells, most commonly in melanoma, travel along the outer (abluminal) surface of blood vessels without invading them
Extracellular migratory metastasis (EVMM)	In this mechanism, cancer cells migrate alongside the outer surfaces of existing anatomical structures, such as blood vessels, nerves, and skin appendages, instead of entering the bloodstream or lymphatic vessels
Extraluminal metastasis route	The term denotes the spreading of cancer cells outside the lumen of the primary organ or beyond the restricted structures of a lymph node (extracapsular spread) into adjacent tissues
Interfacial metastasis route	This pathway involves the spread of cancer cells via tissue interfaces or boundaries, often leading to the direct invasion of adjacent body cavities or anatomical compartments
Intraluminal metastasis route	“Intraluminal metastasis route” refers to the dissemination of cancer cells via blood or lymphoid vessels. Cancer cells intravasate into the vessels, get transported through the fluid flow, extravasate into targeted tissues, and form secondary metastatic tumors
Neurotropism	In cancer, this refers to the capacity of cancer cells to proliferate alongside nerves, infiltrate them, and engage with them. This process is gaining recognition as a key contributor to cancer progression, metastasis, and the development of resistance to treatment
Pericytic mimicry	This is a phenomenon in which cancer cells, especially in melanomas, imitate the behavior of pericytes in that they migrate alongside the abluminal surface of blood vessels and often replace them
Vascular co-option (vasculogenic co-option, vessel co-option)	This is a non-angiogenic mechanism in which cancer cells commandeer and utilize the host’s existing blood vessels to receive oxygen and nutrients rather than stimulating the growth of new vessels. Thus, mixed cancer cell-endothelial cell vessels are formed. Vascular co-option is an important, frequently treatment-resistant pattern of tumor growth in dense organs, such as the brain, liver, and lungs
Vascular mimicry (vessel mimicry)	It is a process in which invasive cancer cells form their own endothelial-independent, 3D vascular-like channels to deliver nutrients to the tumor, bypassing classical angiogenesis. Invasive cancer cells (frequently possessing stem cell-like characteristics) undergo transdifferentiation to resemble endothelial cells and form tubular structures that facilitate the transport of fluid and red blood cells
Transcoelomic spread	In this type of cancer spreading, cancer cells travel through anatomical body cavities, usually the abdominal, chest, or pericardial cavity, to other parts of the body. This metastasis takes place when malignant cancer cells separate from the primary tumor, disperse through fluids, and settle on organ surfaces, like the ovaries, stomach, or lungs

This mechanism, which is not yet fully understood, involves cancer cells mimicking pericytes (a class of cells that encircle blood vessels) and exploiting an embryonic migration mechanism to propagate without invading the lumen of the blood vessel. Therefore, the aim of this review is to recall the alternative extraluminal route of metastasis, to introduce a third interfacial metastasis route, and to analyze these in comparison to the intraluminal metastasis route, highlighting the advantages and disadvantages of each route. Special attention is paid to the mechanobiological aspects and the possible interchangeability of these metastasis pathways, as much as they have been researched. Therefore, this review also highlights the gaps in the mechanobiological analysis of the three metastatic pathways and their possible alternative uses. Nevertheless, the focus remains on comparing the two most important routes of metastasis, intraluminal and extraluminal, as more research results are available on these than on the interfacial metastasis route, also known as vascular co-optation or vascular mimicry. This interfacial pathway has previously been classified as an extraluminal metastasis pathway, but it represents a separate pathway, as cancer cells experience the boundary between the vascular lumen and the extracellular environment. In this interfacial metastasis route, cancer cells can replace endothelial cells and form mixed vessels consisting of cancer cells and endothelial cells (vascular co-option) or completely rebuild vessel-like channels lined solely with cancer cells (vascular mimicry). The molecules and signal transduction pathways involved in these metastasis pathways are briefly outlined as far as they are currently understood. The following section deals with the mechanobiological principles of cancer metastasis that could lead to a better understanding of the process of metastasis and possibly to new insights into cancer therapy.

## 2 Comparison of the mechanobiological principles of the intraluminal and extraluminal metastasis routes

**FIGURE 1:**
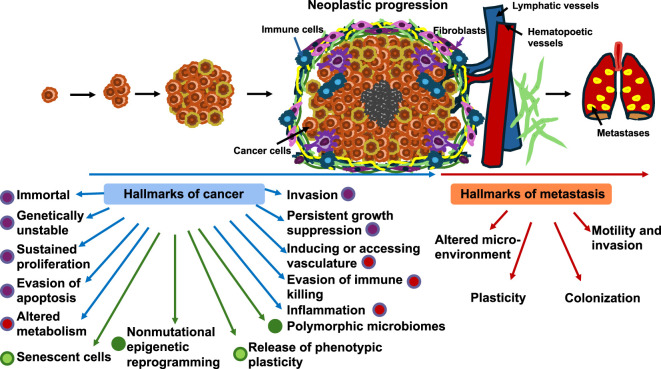
Neoplastic advancement is illustrated by transforming normal cells. Transformed cells can adopt new characteristics to achieve neoplastic potential. This transition is represented by a benign phase; however, not all cells within a neoplasm succeed in obtaining additional traits. The development of cancer/neoplasms is typified through 14 hallmarks of cancer (Hanahan and Weinberg, 2000; 2011; Hanahan, 2022) that comprise six old basic hallmarks introduced in 2000 (purple), four newer hallmarks included in 2011 (red), and four new emerging hallmarks (light green) and enabling characteristics (dark green), such as unlocking phenotypic plasticity, non-mutational epigenetic reprogramming, senescent cells, and polymorphic microbes (Lythgoe et al., 2022). The hallmarks of cancer are overlaid by four “hallmarks of metastasis,” i.e., properties that invasive neoplastic cells need to generate macroscopic secondary tumor masses.

Hallmarks of cancer and newly identified hallmarks of metastatic cancer (Welch and Hurst, 2019) (Figure 1). The trait acquisition can proceed in any sequence, but it is necessary to acquire all neoplastic characteristics for a complete transformation to malignancy. In the same manner, cells must acquire certain properties that overlap with the “hallmarks of cancer” to pass through all stages of the metastasis cascade (Welch and Hurst, 2019). The classical view of cancer metastasis is that each metastatic cell has to pass through a series of consecutive steps, referred to as the metastasis cascade (Fidler, 2003). Nevertheless, these 14 hallmarks of cancer lack the mechanobiological aspect that clearly plays a role (Mierke, 2020). Some mechanobiological effects are exploited by TME, which frequently stiffens around a primary solid tumor, impacting the cancer cells’ own mechanical characteristics (Mierke, 2025c). The process of metastasis must first be examined in depth before the characteristics of metastasis can be exactly defined. The two pathways of cancer metastasis are therefore described below.

### 2.1 Intraluminal metastatic route

**FIGURE 2:**
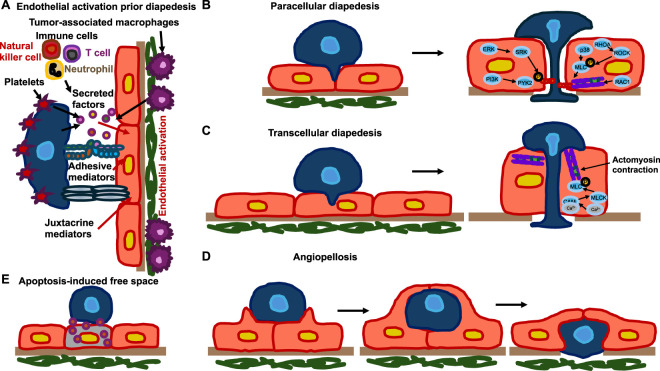
There are different routes for the transmigration of cancer cells through the endothelial cell lining of vessels. **(A)** Diapedesis occurs as a consequence of the activity of at least two regulatory mechanisms: vasoactive factors are released in the metastatic cavity, and direct engagement is achieved between circulating tumor cells (CTCs) (blue) and the adjacent endothelium (red) through adhesion proteins located on the surfaces of these two cell types. Platelets can accumulate surrounding CTCs and create a “protective layer” that shields them from shear stress and immune cells, thereby prolonging their survival. **(B)** Cancer cells can pass between endothelial cells, destroying the intercellular connections. Various signaling cascades engage in this specific event, including PI3K, SRC, ERK, and Rho-GTPases, which trigger actomyosin contraction to widen endothelial openings. Proteins like myosin light chain (MLC) and p38 kinase facilitate the restructuring of the cytoskeleton needed for CTCs to penetrate endothelial boundaries. **(C)** As an alternative route, CTCs traverse the endothelial cells directly, forming a transcellular pore. This process is facilitated by actomyosin contraction and signals involving myosin light chain kinase (MLCK), calmodulin (CaM), and the RhoA signaling pathway, which rearrange the actin cytoskeleton of the endothelium to open the transcellular gate. **(D)** Ultimately, in the extravasation process, CTCs are passively pushed out of the vessel through concerted rearrangement of the microcapillary walls. Endothelial cells build a “pocket” surrounding the cancer cells to push them into the adjacent tissue while maintaining the integrity of the endothelial junctions. **(E)** Activation of endothelial cells can lead to endothelial cell apoptosis. Thus, cancer cells can use the free space to transmigrate. The variety of mechanisms involved in cancer cell transmigration indicates that the endothelium plays an active part in the processes that ultimately trigger metastasis.

The intraluminal metastasis route is the most well-known process of cancer metastasis that is based on the intravascular dissemination of cancer cells. Cancer cells can come into contact with the vascular system either inside or outside the solid primary tumor. It is not clear whether the initial contact of cancer cells with vessels and their basement membrane alters the mode of intravasation. Different models of transendothelial migration have been proposed, such as inter-endothelial (paracellular) transmigration, where cancer cells migrate between endothelial cells, intra-endothelial (transcellular) transmigration, where cancer cells migrate directly through endothelial cells, angiopellosis, which provides an alternative mode of cell intra/extravasation to the mainstream diapedesis hypothesis, and free-space transmigration via the induction of endothelial programmed cell death (apoptosis) (Figure 2) (Peyri et al., 2009; Wittchen, 2009; Allen et al., 2017; Herman et al., 2019). The latter mode of migration is as follows: the cell membrane breaks down in apoptosis, and the connections between cells become untight, leaving a “free space” through which the cell can transit.

After intravasation, cancer cells circulate in the bloodstream and are referred to as circulating tumor cells (CTCs). They face the challenge of resisting the mechanical forces of blood flow. During hematogenous spread, cancer cells are exposed to shear forces that influence their mutual interactions with platelets, neutrophils, and endothelial cells, thereby hampering their survival (Follain et al., 2020). In remote secondary organs, metastatic cancer cells exit the blood vessels, but the overwhelming vast majority of these disseminated cancer cells are either eliminated from cytotoxic lymphocytes or undergo dormancy because of their incapacity to exploit niche assets (Massagué and Obenauf, 2016; Lambert et al., 2017). A tiny subset of these disseminated cancer cells can escape the immune system and adapt to the surrounding microenvironment, eventually developing into deadly metastatic colonies (Fidler, 1995; Vanharanta and Massagué, 2013). Subsequently, CTCs need to exit the blood vessels, which is referred to as the extravasation step, to migrate into the targeted tissues, proliferate, and colonize the metastatic niches through the development of secondary tumors. This process is called cancer metastasis. The underlying mechanobiological principles of these processes are crucial. It is astonishing how well cancer cells from the tissue of the primary tumor can adapt to the new target tissue to survive, proliferate, and give rise to secondary tumors.

Although metastases are the main culprit behind the clinical failure of cancer therapies and mortality, they continue to be poorly understood. In cancer patients, huge numbers of cancer cells are disseminated into the bloodstream every day; nevertheless, investigations in animal models with melanoma imply that fewer than 0.1% of cancer cells finally manage to metastasize (Luzzi et al., 1998). The formation of metastases requires that cancer cells escape their primary tumor, migrate through ECM confinement, circulate in the bloodstream (or lymphoid system), withstand the pressure in the blood vessels, adapt to the new cellular milieu at the secondary tumor site, and evade the lethal battle with immune cells (Massagué and Obenauf, 2016; Maitra, 2019). Hanahan and Weinberg have pointed out that “the activation of invasion and metastasis” is a hallmark of cancer (Hanahan and Weinberg, 2000; 2011). Indeed, the invasion of neighboring tissues and the spread to distant sites to generate metastases continue to be key characteristics of cancer malignancy. While it is still generally accepted that metastases are the main cause of death in over 90% of cancer patients (Sunyer et al., 2012), it has been reported that this percentage is much lower in solid tumors; for example, 66.7% of cancer deaths involved metastases (Dillekås et al., 2019). Therefore, a deeper understanding of the underlying dynamics of this process will contribute to identifying targets for molecular therapeutic interventions that can stall or eventually reduce cancer growth and impair metastasis.

The invasion of individual cancer cells or a cluster of cancer cells into the local parenchyma may occur either actively (cell-autonomously) and/or due to the interstitial fluid pressure arising in the primary solid tumor, which is produced both by the tumor-growing cells and by pressure differences between the leaky blood vessels and the lymph vessels (Piotrowski-Daspit et al., 2016; Stuelten et al., 2018). The development of intratumoral lymphatic vessels promotes lymph node metastasis (Karaman and Detmar, 2014), which, while not fatal, is frequently associated with metastatic progression. Moreover, it can be hypothesized that the presence of lymphoid or blood vessels inside the primary solid cancer is likely to support the intraluminal route of cancer metastasis. Remarkably, recent research has revealed that lymph node metastases can serve as a stage for colonizing the bloodstream (Brown et al., 2018; Pereira et al., 2018). Life-threatening long-distance metastases, by contrast, arise when the cancer spreads through the bloodstream, which is achieved by direct intravasation into the blood vessels. This process can be facilitated by immune cells, such as neutrophils (Harney et al., 2015; Linde et al., 2018), and occurs as either individual cells or collectively invasive cell clusters (Cheung et al., 2013; Gensbittel et al., 2021).

Cancer cells pass into the vascular system through intravasation. As mentioned, they transmigrate into the lumen of blood vessels, cancer cells are referred to as CTCs. In the vessels, they are transported as circulatory cancer cells (CTCs) through the entire system. CTCs can often be detected in the bloodstream of patients suffering from primary tumors; nevertheless, direct histological visualization of cancer cells in vascular tracts is rare (Ribatti and Pezzella, 2022). This discrepancy between the detection methods may be due to the cells’ short retention time in circulation, estimated at a few minutes. In the circulatory system, CTCs are subject to blood flow, shear stress, and/or the activity of immune cells, all of which can kill them. In this context, it is crucial to note that merely 0.1% of intravasated cells survive beyond 24 h, and as little as 0.01% of cancer cells successfully extravasate, invade a secondary tumor site, and subsequently form metastatic foci (Butler and Gullino, 1975; Kienast et al., 2010; Labelle and Hynes, 2012; Ribatti, 2017; Wang et al., 2018; Follain et al., 2020).

**FIGURE 3:**
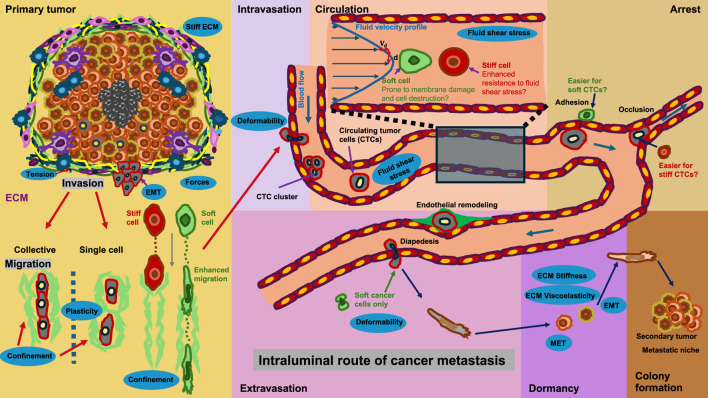
Cancer metastasis can take place via the intraluminal route of cancer metastasis. The steps of the metastatic cascade involve escaping the primary solid tumor and migrating through the ECM confinement. The migration can take place as individual cells or as cancer cell clusters in a collative manner. The cells enter blood vessels through intravasation and can travel as single cells or as CTC clusters. In the bloodstream, they are exposed to fluid shear stress. It remains unclear whether rigid or soft cells are better able to withstand mechanical stress. CTCs can arrest in the vessel lumen and remain in a stalled state. CTCs can also contribute to endothelial remodeling. Cancer cells circulating in the bloodstream need to escape the immune system. CTCs can penetrate the endothelial cell layer and extravasate into the targeted tissue. Thereby, cancer cells need to be able to deform. They migrate through the targeted tissue and can become dormant, where they undergo MET. When conditions in the premetastatic niche become more favorable for cancer cells, they undergo EMT, migrate further into the pre-metastatic niche, multiply, and finally colonize the niche.

Intravasation can occur in two ways: actively and passively (Bockhorn et al., 2007). During passive intravasation, the majority of cells are killed or suffer apoptosis (Bockhorn et al., 2007). It is assumed that these cells are destroyed due to the diminishing supply of nutrients in the hypoxic environment of the primary tumor and the untightened vessels (Bockhorn et al., 2007; Krzyszczyk et al., 2018). In active intravasation, cancer cells travel through the tissue by chemotaxis, following gradients of nutrients and growth factors in the direction of a blood vessel (Stamenkovic, 2000; Roussos et al., 2011). After breaking down the ECM and basement membrane, these cells actively penetrate a blood vessel (Bockhorn et al., 2007). In the bloodstream, these cancer cells possibly bind with blood platelets, enabling the cancer cells to better endure shear forces (Strilic and Offermanns, 2017; Follain et al., 2020). The inducement of EMT in these CTCs enables the reorganization of intermediate filaments to resist this shear force (Xin et al., 2020). In addition, the hypothesis was put forward that softer CTCs can withstand these shear forces more efficiently compared to stiffer CTCs (Mierke, 2025c). Nevertheless, there is still debate about whether cancer cells in the bloodstream need to be stiffer or softer to resist forces and disruptions (Figure 3). Some cancer cells can even evade destruction by the immune system through various mechanisms (Figure 3). When CTCs succeed in evading the immune system and surviving the tug-of-war battle between blood flow forces and adhesion forces (Fan et al., 2016), they ultimately adhere to the vessel walls and become arrested (Osmani et al., 2019). As an alternative scenario, they can also remain adhered regardless of adhesive forces when they are enclosed in small capillaries (Kienast et al., 2010). On rare occasions, particularly in pulmonary metastasis, cancer cells associated with the endothelium are able to proliferate and develop intravascular metastases (Al-Mehdi et al., 2000). In the majority of instances, nevertheless, cancer cells must pass through extravasation, i.e., the process by which trapped cancer cells leave the bloodstream. Several extravasation mechanisms have been documented, such as diapedesis (Strell and Entschladen, 2008) and endothelial restructuring (Lapis et al., 1988). The activation of the transient receptor potential melastatin 7 (TRPM7) channel, facilitated through fluid shear, elicits an extracellular calcium influx, which subsequently activates the RhoA/myosin II and calmodulin/IQGAP1/Cdc42 signal transduction routes and reverse the direction of migration in a concerted manner, thus preventing shear stress (Yankaskas et al., 2021). Cells that exhibit increased shear sensitivity because of higher TRPM7 activity extravasate more slowly and form fewer invasive metastatic lesions. Consequently, these results yield a mechanistic interface for the involvement of shear stress and its sensor, TRPM7, in the extravasation of cancer cells (Yankaskas et al., 2021). In addition, CTCs can mechanically adapt, for instance, they can stiffen due to elevated fluid shear stress and are hence protected through formin and myosin II activity against destruction (Moose et al., 2020). This departure from the bloodstream ultimately enables cancer cells to access the stroma/parenchyma of neighboring organs, where they can either multiply and develop metastases when the microenvironment of the target organ is favorable (Peinado et al., 2017) or stay in a quiescent state pending further development of an environment that enables them to prosper (Nguyen et al., 2009; Aguirre-Ghiso, 2018) (Figure 3).

The liberation of soluble molecules like VEGF, IL-10, TGF-β, prostaglandin E, and Fas by cancer cells leads to the development of an immunosuppressive milieu and, consequently, chronic inflammation (Kim et al., 2007; Schreiber et al., 2011; DuPage et al., 2012; Matsushita et al., 2012; Joseph et al., 2021; Tripathi et al., 2025). For instance, VEGF release results in the recruitment of immature dendritic cells and macrophages (Gabrilovich et al., 1996; Schoppmann et al., 2002; Mierke, 2024c). Tumor-associated dendritic cells and tumor-associated macrophages (TAMs) repress the capacity of mature dendritic cells and macrophages to eradicate cancer cells through inhibiting the activation of T-cells and preventing phagocytosis (Kim et al., 2007; Wang J. et al., 2019). Moreover, antigen presentation, especially by the major histocompatibility complex (MHC), is reduced on the surface of cancer cells, which helps them evade immune surveillance (Teng et al., 2013). During tumor advancement, these components of the ECM environment are capable of experiencing modifications in both structure and function, leading to solid stresses, which are mechanical stresses resulting from the structural elements of the primary solid tumor (De Visser et al., 2006; Pickup et al., 2014; Nyga et al., 2021; Angeli et al., 2025). Cancer cells or activated fibroblasts cause the ECM to change by stretching collagen fibers, which, along with the fast growth of the tumor within the limited area of the host tumor, leads to the development of solid stress. Moreover, the activation of fibroblasts leads to the release of TGF-β (Casey et al., 2008; Costea et al., 2013; Papageorgis and Stylianopoulos, 2015; Afik et al., 2016; Pankova et al., 2016), resulting in an excess of ECM constituents, such as collagen, fibronectin, and hyaluronic acid, in addition to enhanced levels of collagen crosslinking. TGF-β overexpression is linked to elevated elongation of cancer-associated fibroblasts (CAFs), spreading of cancer cells, development of lamellipodia, and the invasion of cancer spheroids (Stylianou et al., 2018). TGF-β actions make the ECM stiffer, thereby affecting cancer cell performance (Kalli and Stylianopoulos, 2024). Heightened tissue stiffness also induces fibroblast activation and subsequent generation of α-smooth muscle actin (α-SMA)-positive myofibroblasts, which are referred to as CAFs (Stylianou et al., 2019). This leads to a positive feedback cycle in which increased activation of CAFs fuels ECM generation, crosslinking of ECM, and contractility of myofibroblasts, causing the tissue to become even stiffer (Tomasek et al., 2002). While myofibroblast-like CAFs are generally considered to facilitate cancer advancement (Erez et al., 2010; Su et al., 2018), evidence has demonstrated that they may also suppress tumors according to the specific fibroblast type and tissue of origin (Rhim et al., 2014; Özdemir et al., 2014). For example, hyaluronan, which is secreted by CAFs, has been seen to enhance tumor growth, while collagen I, which is also released by CAFs, has been shown to inhibit tumor growth (Bhattacharjee et al., 2021).

Most intriguingly, the specific makeup of immune cells within the TME can either encourage or impair the restructuring of the ECM and, thereby, impact the stiffness of the tissue. For example, in mouse breast tumors, increased matrix stiffness directly coincided with the deposition of M2-like TAMs (Taufalele et al., 2023), whereas other immune cells, such as cytotoxic T cells, may be involved in breaking down the ECM. As a result, a collagen-rich, stiff ECM restricts the penetration of T cells and attenuates their cytotoxic ability. The heightened rigidity provides a physical barrier to T cell infiltration of the tumor and also reduces their capacity to efficiently attack and eliminate cancer cells (Reis-Sobreiro et al., 2021; Marofi et al., 2022; Vignali et al., 2023).

Another protective mechanism for cancer cells involves the interference with other cell types within blood vessels. For example, platelet cell coating can protect CTCs against natural killer cells and T cells, and platelets can confer MHC to cancer cells, thereby deceiving the immune system (Lou et al., 2015). Immune checkpoint inhibition treatments can bypass immune evasion through augmenting T-cell-driven destruction of cancer cells, like melanoma cells, by interfering with the PD-1 immune checkpoint using intermittent antibodies (Brahmer et al., 2010). Localization of YAP conveys the mechanical adaptation in human cancer cells through extravasation *in vivo* (So et al., 2023). This suggests that cancer cells that have spread can continue to mutate with a seamless spectrum of mechanical phenotypes under the influence of YAP-induced mechanosensory mechanisms of hydrodynamic flow (Mierke, 2019; So et al., 2023). At each step of this metastasis cascade, cancer cells must adjust to the biophysical characteristics of their surrounding environment, traveling through a complicated network of forces exerted by the ECM, fluid flow, and passive or active engagements with other cells. In recent years, it has become apparent that intrinsic cellular mechanical characteristics, such as cytoskeletal stiffness and membrane tension, are of key importance to this process (Mierke, 2019; 2020; Er et al., 2022). Despite this, there is growing indication that some cancers, particularly melanomas and carcinomas, can employ various mechanisms for growth and propagation, for instance, the extraluminal metastasis pathway, which is discussed below.

Building on the context of the mechanical microenvironments of cancers and metastasis routes, the direct influence of mechanical elements on these events is presented here, with a concentration on the intraluminal route (intravasation/circulation/extravasation) and the accompanying mechanical features. First, there are mechanical forces that promote the intravasation process. Mechanical forces include solid stress and interstitial fluid pressure (IFP). Compressive stress arises when a primary tumor undergoes accelerated growth. As a result, a high-pressure surrounding environment is built up. This compressive stress pushes the cancer cells away from the center of the cancer itself and toward the perimeter, pressing them directly into the walls of the cancer-related blood vessels. Vascular compression develops in the primary cancer. In addition, leaky neovascularization occurs. The high interstitial pressure causes compression of blood and lymph vessels, whereas tumor-induced blood vessels are frequently compromised with tortuosity and leakage. This leads to increased permeability and reduced perfusion, thereby facilitating the ability of cancer cells to bypass the endothelial barrier through shear forces or by exploiting small openings in the blood vessels. In addition, mechanical stress and the associated biochemical restructuring result in cancer cells shedding their adhesion with adjacent cells through a decrease in E-cadherin, which facilitates individual or collective invasion (the “unjamming transition”) into the lumen of blood vessels. Second, CTCs are subject to physical challenges. When they are in the lumen of the bloodstream or lymphatic vessels, the mechanical environment changes from high pressure to high speed because of fluid shear stress (FSS). High shear stresses in the bloodstream can destroy cancer cells. Cancer cells display reduced stiffness (being more malleable) and increased intracellular viscosity for the purpose of resisting these forces and thereby ensuring survival in this specific mechanical environment. In addition, there is a size constraint for the microvessels, as cancer cells with a diameter of approximately 15–20 µm are usually larger compared to capillaries, with a diameter of 3–12 µm. This physical incompatibility forces CTCs to change shape in extreme ways (squeezing) to pass through, which functions similarly to a filter, so that only highly deformable cells can remain viable. Additionally, cancer cells are protected from being destroyed by forming clusters. For instance, when cancer cells travel as clusters rather than individual cells, they can line up in a single chain to squeeze through narrow capillaries, enabling them to endure the hydrodynamic shear forces that may otherwise break them apart. Third, mechanical forces come into play during the extravasation phase. The physical confinement (occlusion) of larger cancer cells within small capillaries, which is referred to as mechanical entrapment, impedes their movement. This physical deceleration enables adhesion and subsequent endothelial transmigration (extravasation) of the cancer cells into the distantly located tissue. In addition, cancer cells can be actively or passively reshaped. Cancer cells capable of dynamically adjusting their mechanical characteristics, such as by altering their cytoskeleton, are capable of extruding themselves from the bloodstream in a more efficient manner. For instance, they can wriggle through openings in tissue or become spindle-shaped to pass through dense structures, such as bone tissue.

### 2.2 Extraluminal metastatic route

At first glance, the metastatic cascade appears to be clearly outlined, but upon closer inspection, details are missing, such as the alternative extraluminal route of cancer metastasis. The first appearance of this route was described for melanoma cells by Lugassy and Barnhill in their review in 2007 (Lugassy et al., 1998; Lugassy and Barnhill, 2007). This process, in which melanoma cells imitate pericytes and propagate alongside the abluminal (outermost) surfaces of microvessels, is considered an integral part of melanoma metastasis. While the intraluminal metastasis route has been identified in all cancer types, the extraluminal route has only been detected in specific cancer types using various techniques. The occurrence of intraluminal and extraluminal metastasis routes in different cancer types has been listed in Table 2.

**TABLE 2:** The two routes of cancer metastasis can occur in different types of cancer.

Cancer type	Intraluminal metastasis route	Extraluminal metastasis route
Breast cancer	Vascular metastasis (Çalım Gürbüz et al., 2024)	Çalım Gürbüz et al. (2024)
Glioma	Vascular metastasis (Lugassy et al., 2002)	The GL26 murine glioma cell line was analyzed in an *in vivo* murine brain tumor model. In addition, *in vitro* analysis was carried out through the co-culture of endothelial cells that created capillary-like structures with cancer cells (Lugassy et al., 2002)
Glioma	Vascular metastasis (Lugassy et al., 2002)	In in vitro organotypic slice tissues, in an *in vivo* mouse model, and in clinical observations (Zagzag et al., 2008; Winkler et al., 2009; Montana and Sontheimer, 2011; Watkins et al., 2014; Alieva et al., 2019)
Gynecological (tubo-ovarian and endometrial) carcinosarcoma	Vascular metastasis (Dyke et al., 2014)	Observed in malignant cancer cells, such as tubo-ovarian and endometrial carcinosarcomas, which exhibited EVMM, appeared sarcomatoid, and were abluminally dispersed and partially or completely encircled the endothelium. Affected vessels usually show fibrin plaques on the vessel wall. Immunohistochemical analysis demonstrated that perivascular malignant cells exhibited more persistent SMA and laminin immunoreactivity compared to nonvascular tumor entities (Dyke et al., 2014)
Melanoma	Vascular metastasis (Lugassy and Barnhill, 2007)	B16 melanoma cells (Lugassy et al., 1998)
Melanoma	Vascular metastasis (Moy et al., 2019)	Retrospective study of 179 primary cutaneous melanomas (Moy et al., 2019)
Melanoma	Vascular metastasis (Rodewald et al., 2019)	Eight autopsy cases (Rodewald et al., 2019)
Melanoma	Vascular metastasis (Wilmott et al., 2012)	Angiotropism in patient samples (Wilmott et al., 2012)
Melanoma	Vascular metastasis (Bentolila et al., 2016)	Angiotropism/Vascular co-option in patient samples (Bentolila et al., 2016)
Melanoma/uveal melanoma	Vascular metastasis (Barnhill and Lugassy, 2004)	*In vitro* co-culture system, *in vivo* CAM assay, murine and zebrafish models, and clinical observations (Barnhill and Lugassy, 2004; Lugassy et al., 2007 ; 2013; Fornabaio et al., 2018)
Pancreatic cancer	Vascular metastasis (Levy et al., 2009)	In clinal observations (Levy et al., 2009)
Prostate cancer	Vascular metastasis (Lugassy et al., 2005)	*In vitro*, PC-3 cells disseminated alongside the outer face of the vascular tubules. *In vivo*, PC-3 cells established a band around some vessels a few millimeters outside the primary tumor, demonstrating angiotropism. The histopathology of the CAM revealed the perivascular distribution of cancer cells and the complete lack of cancer cells within the vascular lumen (Lugassy et al., 2005)
Sarcomatoid squamous cell carcinoma	​	In clinical observations (Rodewald et al., 2019)
Syringomatous carcinoma	​	In clinical observations (Katayama et al., 2017)
Well-differentiated liposarcoma	​	In clinical observations (Shen et al., 2016)

**FIGURE 4:**
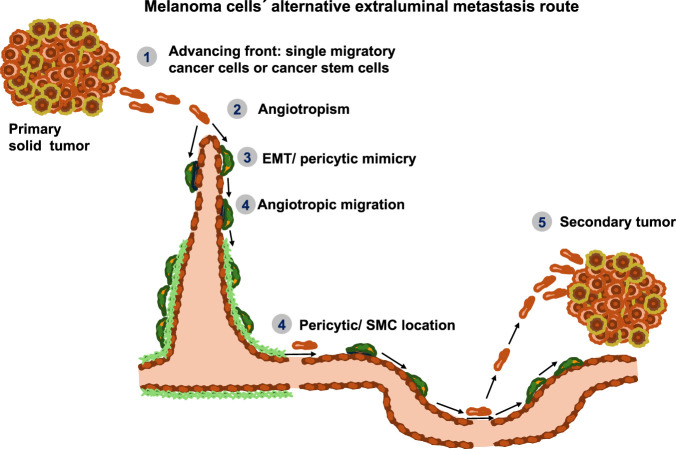
The alternative route of metastasis that cancer cells, such as melanoma cells, can use. First, cancer cells, or possibly cancer stem cells, can spread at the front edge of the primary tumor. Second, they migrate toward blood vessels and move along their structures, a process known as angiotropism. Third, metastatic cancer cells can undergo EMT and/or perform pericytic mimicry. Fourth, angiotropic migration takes place. Cancer cells can move either to the location of the pericytes or the smooth muscle cells that line the blood vessels to mimic their shape and possibly also some of their function. Fifth, cancer cells can migrate away from blood vessels toward their pre-metastatic niche. There, they begin to multiply and form secondary tumors.

In melanoma specimens, the presence of individual cancer cells or cell aggregates in the vicinity of vessels on their outer surface has been identified by pathologists. Lugassy and Barnhill characterized the movement of melanoma cells alongside the abluminal surface of vessels through a process referred to as “extravascular migratory metastasis” (EVMM) (Figure 4) (Lugassy et al., 1998).

Angiotropism has been defined as a histopathological marker of EVMM and is characterized by clusters of melanoma cells that appear on the outer surface of blood vessels or lymph vessels within 1–2 mm of the progressing primary tumor’s leading edge (Barnhill et al., 2002; Lugassy and Barnhill, 2007). Moreover, it can even be used to predict the risk of cancer metastasis (Barnhill et al., 2002). It can therefore be said that the angiotropic process represents the movement of cancer cells on the abluminal surface of vessels. Cancer cells that adopt the typical shapes and locations of pericytes can travel outside of blood vessels and revert to a neural crest cell migration phenotype (Landsberg et al., 2016). Based on this concept, melanoma cells that are tightly connected to the endothelium at a pericytic area, and are usually found at the leading edge of the cancer without signs of intravasation, are classified as angiotropic melanoma cells (Barnhill and Lugassy, 2004; Lugassy et al., 2020). Histological analysis has shown that both micro- and macro-vessels can be impacted through angiotropic events, whereas neovascularization or stabilized vessels have not yet been conclusively demonstrated. In this system, the endothelial cells exhibit no evidence of physiological injury or intravasation. Alongside classic metastatic spread, the out-of-vessel migration of cancer cells has been proven as EVMM for certain types of cancer (see Table 2).

**FIGURE 5:**
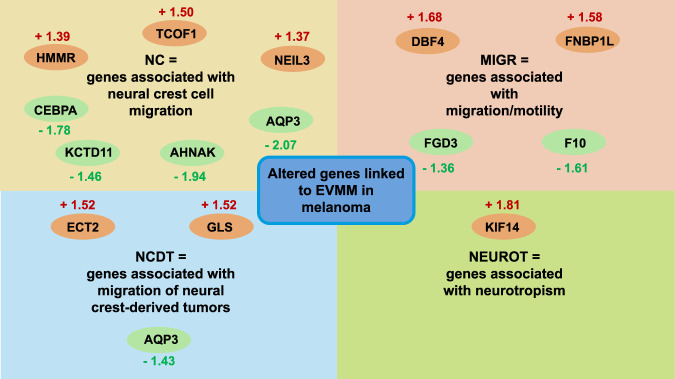
Identified genes modulated in melanoma patients that are linked to EVMM (Lugassy et al., 2011).

How important is the extraluminal route of metastasis? The concept that cancer cells metastasize exclusively via circulation within the vascular tract has been challenged by the emergence of angiotropism, pericytic mimicry, and EVMM (Table 1). EVMM constitutes a new paradigm for the spread and dissemination of cancer and, thus, for its malignant advancement—the metastasis of cancer. EVMM can therefore be regarded as an effective alternative to tumor spread. During cancer metastasis, cancer cells use embryonic mechanisms to attach themselves to the abluminal surfaces of blood vessels in a process known as angiotropism. In this way, cancer cells can disseminate through nonstop migration, where they compete with pericytes and even steal their place, which is termed pericyte mimicry. EVMM and pericytic mimicry, which are frequently observed in melanomas (Barnhill and Lugassy, 2004), have been reported in a number of tumors, among them squamous cell carcinomas of the skin, adenocarcinomas of the prostate and pancreas, carcinosarcomas of the ovary and endometrium, gliomas, liposarcomas, and glioblastomas (Table 2) (Lugassy et al., 2005; Cheng et al., 2013; Rustagi et al., 2019). Throughout EVMM, the localization of melanoma cells in the perivascular space has been linked to the development of stem cell-like plasticity. In addition, these cancer cells express specific pericytic markers, which comprise PDGFRβ, CD146, CD44, CD73, CD105, and CD144 (Lugassy et al., 2014). These findings confirm the characteristics of glioblastomas, in which EVMM can be seen and cancer stem cells make up to 80% of the pericytic compartment. Despite these similarities, additional research is necessary to gain insight into whether pericytes in glioblastomas and melanomas have similar migration and metastasis capabilities and if they exhibit a specific function in vascular stabilization. A previous study indicates that EVMM is present in up to 37% of melanoma incidences (Moy et al., 2019). EVMM is thus likely to be a widespread phenomenon that has been largely overlooked because of technical constraints. In addition, it has been noted that advanced melanoma patients with negative sentinel lymph nodes exhibit progressive disease in both the sentinel basin and at distant locations. Thus, the hypothesis was put forward that EVMM, instead of hematogenous dissemination, could be accountable for the seen progressive disease affecting a single organ. In support of this hypothesis, it is essential to identify the mechanisms of EVMM action. For example, the differential analysis conducted between angiotropic melanomas and non-angiotropic melanomas identified 15 critical genes that are implicated in the regulation of EVMM (Figure 5) (Lugassy et al., 2011).

Kinesin family member 14 (KIF14), epithelial cell transformation sequence 2 oncogene (ECT2), and hyaluronan-mediated motility receptor (HMMR) are among them and appear to be involved in cytokinesis. Moreover, co-culture studies revealed that the engagement of angiotropic melanoma cells with the abluminal vessel surface enhances the differential expression of genes associated with cell migration, such as CCL2, ICAM1, and IL6, cancer progression, such as CCL2, ICAM1, SELE, TRAF1, IL6, SERPINB2, and CXCL6, EMT, such as CCL2 and IL6, stem cell-like properties, such as CCL2, PDGFB, EVX1, and CFDP1, and pericytic recruitment, such as PDGFB (Lugassy et al., 2013).

As this new mechanism finally leads to metastatic spread of cancer cells, it can be seen as an alternative route for cancer metastasis. Consequently, the question arises as to whether the metastatic pathway selection offers a strategy to avoid being attacked by mechanical cues of the circulation, along with immunological cues, such as immune cells, including natural killer cells and T lymphocytes (Gu et al., 2024). Moreover, the following discussion will examine whether further subdivision into luminal and extraluminal pathways can remedy this situation.

## 3 Is the extraluminal metastasis route an alternative pathway for cancer metastasis?

Intravascular spread of cancer cells is still considered the established pathway for metastasis. In addition, the angiogenic mechanism is an integral part of the metastatic process. The vascular bed inside the cancer is the main way that cancer cells leave the primary cancer and pass into the bloodstream. In many cancers, vascular and lymphatic coverage is a prognostic marker for metastatic capacity, with highly vascularized primary cancers exhibiting a higher rate of metastasis than those with poor vascularization (Fernández-Cortés et al., 2019). The vascular network serves as a physical and mechanical barrier preventing cancer cells from entering circulation, thereby influencing their destiny and the new target tissue.

### 3.1 Subdivision of cancer metastasis into two major routes, the intraluminal and extraluminal pathways, and emergence of a third interfacial route

Angiotropism, pericytic mimicry, and EVMM, nevertheless, have cast doubt on the hypothesis that cancer cells metastasize exclusively via blood circulation inside vessels (Table 1). All of these processes employ an extraluminal metastasis route. This new metastatic spread and metastasis model proposes that metastatic cells utilize embryonic mechanisms to adhere to the abluminal vascular surfaces (angiotropism) and propagate through sustained migration, thereby outcompeting and exchanging pericytes, a process termed pericytic mimicry (Donnem et al., 2013; Kuczynski et al., 2019). This biological mechanism resembles the migration of cancer cells along anatomical structures, such as nerves and skin appendages, and has been previously identified (Marchesi et al., 2010). EVMM is a completely extravascular event that does not involve invading (intravasation) of blood vessels. Pericyte mimicry and EVMM have primarily been investigated in melanoma, but they also arise in certain other types of cancer (Table 2). Pericyte mimicry and EVMM seem to be a regression to a specialized developmental program originating from embryogenesis (Lugassy et al., 2011). Many analogies exist between embryogenesis and cancer progression, among them the significant contribution of laminin, the epithelial-mesenchymal transition, and the resetting of embryonic cues through cancer cells. In addition, there is no blood flow in the first trimester of embryogenesis, even though extensive cell migration to distant locations and the formation of organs and tissues take place at this time. Embryonic migration is consequently a nonstop extravascular migration, as are pericytic mimicry and EVMM, which underpins the idea that these embryonic migration phenomena evidently reappear aberrantly during metastasis. Ultimately, the perivascular positioning of cancer cells inextricably connects pericytic mimicry with vascular co-option (Table 1). Collectively, these two new modalities could have a profound impact on the development of new, efficacious metastatic therapies (Ingangi et al., 2019). These therapies must take into account the different pathways of cancer metastasis and induce a transition from the extraluminal to the intraluminal metastasis pathway if the metastasis therapies are based on blocking the intraluminal metastasis pathway. Another approach appears to be the targeted manipulation of embryonic migration factors that are involved in cancer metastasis and could be particularly important for pericytic mimicry or EVMM. This approach could be combined with the blocking of the intraluminal metastasis route.

How are cancer cells capable of interacting with blood vessels? Intravascular spread of cancer is generally accepted as the primary, if not the sole, pathway of metastasis. The interactions between a primary tumor and its vascular system were for many years considered to be primarily concerned with, firstly, the angiogenesis necessary for the growth of tumors at the primary and secondary sites and, secondly, the intravascular dissemination of cancer cells to the sites of metastasis. Nevertheless, the specific mechanisms of tumor growth and cancer cell migration, especially the role played by cancer vessels in these processes, are the subject of ongoing research (Chitty et al., 2018). While cancer cells in direct physical engagement with the abluminal surface of blood vessels were assumed to be undergoing intravasation, evidence emerged that such interactions between blood vessels and cancers potentially represent a distinct biological process. Specifically, the efforts of separate research groups investigating the biological properties of cancer cells distributed alongside the abluminal vascular surface have identified two new avenues of cancer research. The first is vascular co-option (synonymously termed vessel co-option) and vascular mimicry, and the second is angiotropism, pericytic mimicry, and EVMM. These two tightly linked topics pose crucial challenges to two distinct but interconnected facets of cancer biology. It is notable that the literature on the angiogenesis of cancers overlooks a long-standing but recurring line of research pointing to the possibility that cancers may instead grow through capturing existing, surrounding non-malignant blood vessels. Cancer cells can multiply along the blood vessels of adjacent healthy tissue. These cancer cells migrate alongside the abluminal surface of pre-existing vessels (the extraluminal metastasis route), which frequently results in the removal of noncancerous cells (the interfacial metastasis route). In this way, these cancer cells can invade normal vessels and ultimately take them over rather than promoting the stimulation of new abnormal sprouting (angiogenesis). This phenomenon, referred to as vascular co-option, is an underestimated mechanism of tumor angiogenesis that can impact the progression of the disease, its metastatic spread, and the responsiveness to therapy. The concept of vascular co-option, developed by Pezzella and coworkers and discussed in Kuczynski et al. (2019), has highlighted the essential role of the non-angiogenic phenotype in the initiation and progression of cancer. Moreover, vascular co-option questions the long-standing paradigm that all tumor growth relies on angiogenesis (Weidner et al., 1991) and is considered a non-angiogenic mechanism by which cancers capture existing vasculature (Kuczynski et al., 2019). Vascular co-option has been demonstrated at both primary and metastatic locations without compromising the “exclusive” intravascular pathway of spread between primary and secondary locations. Vascular co-optation is exploited across a broad variety of human tumors arising in multiple tissues, such as the brain, liver (Frentzas et al., 2016), lungs, and lymph nodes. Vascular co-option influences disease progression and resistance to oncological treatments and provides a valid therapeutic goal. Another mechanism is vascular mimicry. Vascular mimicry (synonymous to vascular mimicry or vascular mimicry) and vascular co-option are mechanisms that enable tumors to grow and survive independently of classical angiogenesis. In vascular co-option, cancer cells capture existing blood vessels from the patient. In vascular mimicry, cancer cells build new fluid channels themselves (Wei et al., 2021). Aggressive cancer cells (frequently stem cell-like) generate their own operational blood vessels independently of endothelial cells (Wei et al., 2021). These channels are generally CD31-negative (without endothelial cell markers). Nevertheless, they exhibit periodic acid-Schiff (PAS)-positive staining, which identifies carbohydrate-rich substances, such as glycogen, glycoproteins, and mucins, and indicates the basement membrane. In addition, these channels are encircled with cancer cells. The mechanism of vascular co-option is induced by hypoxia and the elevated plasticity of cancer cells. Both are non-angiogenic, which implies that they are not reliant on vascular endothelial growth factor (VEGF) signaling to establish new blood vessels. Both mechanisms are key drivers of resistance to anti-angiogenic therapeutic approaches, as the tumor responds by leveraging pre-existing or self-generated channels. Further research efforts are required to target both vascular co-option and vascular mimicry, as conventional anti-angiogenic inhibitors are frequently inadequate in counteracting these mechanisms.

**FIGURE 6:**
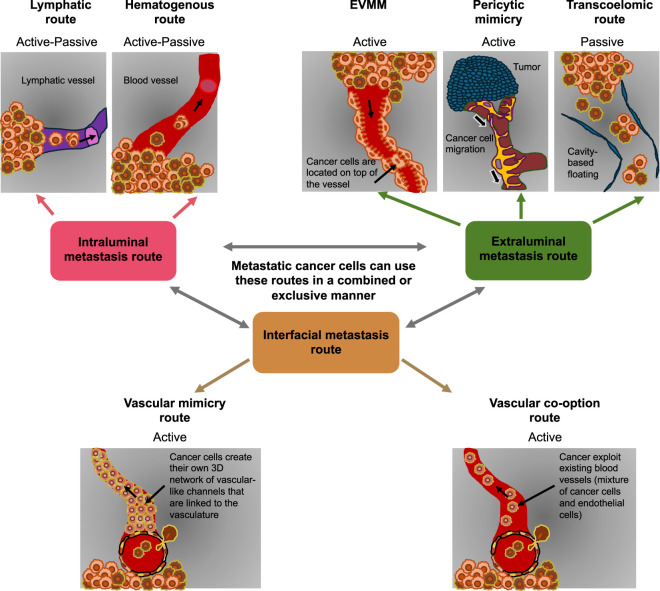
There are two major routes of cancer metastasis and another third interfacial metastasis route. The two major routes of metastasis consist of active and passive portions. The two subroutes of intraluminal metastasis, the hematogenous and lymphatic routes, contain both active and passive parts. In the lymphatic metastasis route, metastatic cancer cells that have dissociated from the primary tumor destroy the basal lamina (basement membrane) of the lymphatic vessels and penetrate them (intravasation). Metastatic cancer cells gain access to the lymphatic network via active migration, travel up the lymphatic flow, and populate the lymph nodes by colony formation and colonize other organs. Through the hematogenous route, metastatic cancer cells that have dislodged from the primary tumor converge on capillaries or angiogenic blood vessels, destroy the basement membrane, penetrate the endothelium, and enter the bloodstream as individual cells or in small groups. Ultimately, they settle in susceptible distant organs to generate secondary tumors. The extraluminal metastasis route includes extravascular migratory metastasis (EVMM), pericytic mimicry, and the transcoelomic route. In contrast to EVMM and pericytic mimicry, which are active movements, the transcoelomic route appears to be passive. On the EVMM pathway, metastatic cancer cells that have separated from the primary tumor move toward capillaries or angiogenic vasculature. When they are attached to a vessel, the cells travel alongside the abluminal side but without intravasating, breaking down the basement membrane, or modifying the vascular architecture. Metastatic cancer cells that have disintegrated or separated from the tumor persist in the cavities as individual cells or clusters of cells via the transcoelomic route. Cancer cells disseminate into cavities in the body by infiltrating the surface of the peritoneum, pleural cavity, pericardial cavity, or subarachnoid space. In these cavities, metastatic cancer cells multiply in suspension, produce ascites, and/or may adhere to different targeted tissues. The interfacial metastasis route is the migration of cancer cells at the boundary of the intraluminal and extraluminal routes. There are two distinct types of interfacial metastasis. First, in vascular mimicry (synonymously referred to as vasculogenic mimicry), metastatic cancer cells or cancer stem cells (CSCs), which undergo epithelial-to-endothelial transition (EET), can create vascular tubules that are linked to the vasculature of the tumor when under hypoxic pressure. This type of cell is referred to as a tumor endothelial cell and mimics endothelial cells. These leaky structures supply the cancer with nutrients and oxygen and encourage the dissemination of metastatic cells. Thereby, these tumor endothelial cells are located at the interface between the lumen of the vessels and the extracellular environment. Consequently, cancer cells can utilize this new interfacial metastasis pathway, as it is located at the boundary between an intraluminal metastasis pathway and an extraluminal metastasis pathway. Second, cancer cells can carry out vascular co-option and hijack existing blood vessels, whereby a mixture of endothelial cells and cancer cells creates these vessels. During vascular co-option, cancer cells are located at the interface between the lumen of the vessels and the extracellular microenvironment. This occurs because cancer cells break away from the primary tumor, drift in the fluid of the body cavity, adhere to the surfaces of other organs inside this body cavity, and proliferate there. This process often takes place in advanced colon, lung, ovarian, and stomach cancers (Van Baal et al., 2017; Ritch and Telleria, 2022).

Investigations into angiotropism, pericytic mimicry, and EVMM have challenged the idea that all cancer cells metastasize strictly within vascular lumina (Figure 6). Angiotropism, pericytic mimicry, and EVMM represent three mutually complementary concepts that relate to an embryologically deduced pathway of ongoing extravascular cancer cell migration without entering vascular conduits (see Figure 4). All represent extraluminal metastatic routes (Figure 6). Besides moving along vascular channels, like with angiotropism and pericytic mimicry, EVMM can also appear alongside other anatomical structures, like nerves, in neurotropism (Table 1) (Graham et al., 1994).

Angiotropism has been seen in both primary and metastatic cancers alongside existing blood vessels, as in vascular co-option, but also alongside new vessels in experimental models. In contrast to vascular co-option, angiotropism also involves the continuous migration of cancer cells in the absence of intra- or extravasation. Angiotropic cancer cells self-localize alongside the abluminal vascular sites where they compete with and mimic pericytes (or other perivascular mesenchymal stem cells). In the first step, cancer cells are actively attracted to the abluminal surface of the vessel and substitute the pericytes. In the second step, the cancer cells move alongside the abluminal surface. Nevertheless, unlike pericytes, cancer cells fail to consolidate either the neovasculature or the vascular basement membrane matrix. Pericytic mimicry permits cancer cells to propagate alongside blood vessels, receiving all necessary nourishment and oxygen, thereby preventing the deterioration of cancer cells experienced during dissemination through the bloodstream (Weidner et al., 1993; Prakash et al., 2021). Transcoelomic spread is a form of cancer metastasis in which cancer cells disseminate via a cavity in the body, such as the peritoneal or pleural cavity (Table 1) (Ritch and Telleria, 2022).

The perivascular site of cancer cells inextricably connects pericytic mimicry with vascular co-option. In pericyte mimicry, cancer cells acquire a migratory phenotype; that is, they migrate to secondary sites. In contrast, cancer cells acquire an invasive/proliferative phenotype for cancer growth in vascular co-option. Unlike pericytes, cancer cells do not stabilize neovasculature or the vascular basement membrane architecture. Cancer cells can use pericyte mimicry to move alongside blood vessels and obtain the nutrients and oxygen they require. The avascular movement of cancer cells helps them avoid cancer death induced by intra- or extravasation of blood vessels, which in turn is caused by these two steps of the vascular metastasis cascade or the high forces resulting from vascular flow. Some questions remain unanswered: Can angiotropism also be observed as a similar phenomenon in lymphatic vessels? Is angiotropism evident in organs/tissues that are devoid of pericyte lining of blood vessels? Can cancer cells replace other cells instead of pericytes, such as smooth muscle cells? It is possible that cancer cells can replace the smooth muscle cells that surround blood vessels. Pursuing this approach further could result in angiotropism even without pericytes. There appears to be nothing to oppose angiotropism in lymph vessels. Nevertheless, there is still considerable research work to be done in this area before these questions can be answered comprehensively.

In gliomas, bradykinin, CXCR4, and Wnt7 have been found to promote the dissemination of glioma cells along blood vessels (Montana and Sontheimer, 2011; Yadav et al., 2016; Griveau et al., 2018). In addition, it has been suggested that adhesion molecules, metabolic signals, and signaling routes associated with cancer cell motility promote angiotropism or vessel co-option in several types of cancer (Valiente et al., 2014; Yao et al., 2018). Cancer cells also utilize contact guidance and chemotactic signals to arrive at their final metastatic destinations (Gandalovičová et al., 2016). Angiotropism has primarily been investigated and established as a prognostic determinant for the metastasis and survival of melanoma cells in cutaneous melanoma (CM) and uveal melanoma (UM) (Barnhill et al., 2002; 2016; 2017; 2018; Barnhill and Lugassy, 2004; Van Es et al., 2008; Bald et al., 2014; Lugassy et al., 2014). Angiotropic melanoma cells not only originate as neural crest cells; they also exhibit the same specialized characteristics and mechanisms observed in migrating neural crest cells in the embryogenesis (Lee and R Hall, 2016). Previous molecular investigations have been conducted on angiotropism in melanoma (Lugassy et al., 2020). Cell motility/migration, embryonic/cancer stem cells, inflammation, and the pericyte markers are especially germane to angiotropic melanoma. Since there are these different pathways of cancer metastasis, it remains to be discussed whether cancer cells can switch between these pathways. Nevertheless, it must be investigated how cancer cells can select a specific metastasis pathway.

### 3.2 What factors influence the decision of which route to take?

**FIGURE 7:**
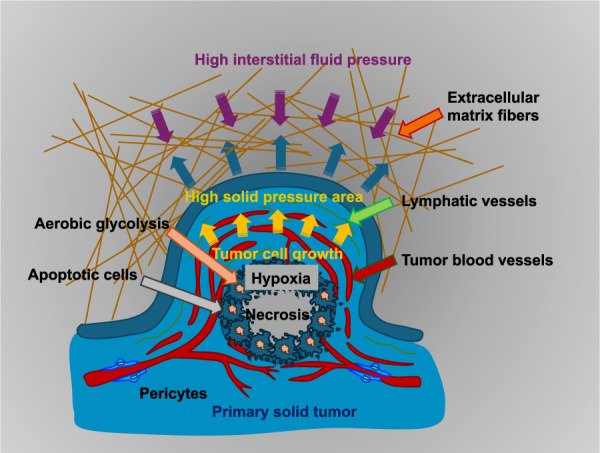
External cues on the primary tumor act on the cancer cells inside the tumor mass. In the center of the tumor are necrotic cells. At the border of the necrotic plaque are apoptotic cancer cells. The primary solid tumor induces neovascularization, as inside its core are hypoxic conditions.

The decision whether cancer metastasis occurs intraluminally or extraluminally depends on important mechanobiological principles, including cell-matrix interactions, cellular forces and motility, the TME, interaction with the endothelial cell lining of blood vessels, EMT, and/or the jamming-to-unjamming transition (Mierke, 2025a), as well as mechanosensing and mechanotransduction. Regarding the mechanobiological principle of cell-matrix interaction during malignant progression, cancer cells use special proteins referred to as integrins to communicate with the ECM, a complex network of proteins and molecules located outside the cells. Several integrins can selectively bind to a single ECM protein ligand, for instance, the integrin α5β1 can recognize primarily fibronectin, while other integrins can bind to different ligands, for instance, integrin αvβ3 connects to vitronectin, fibrinogen, fibronectin, collagen (both denatured or proteolyzed), and various other matrix proteins. These integrins are recognized as mechanosensory molecules that transduce mechanical signals and can transmit cellular forces to the microenvironment (Levental et al., 2009; Mierke, 2020; 2024b; Katoh, 2025). Some integrins, such as αvβ3, α5β1, and αIIbβ3, detect the tripeptide Arg–Gly–Asp (RGD), while several other integrins, such as α4β1, detect alternative short peptide sequences, such as EILDV and REDV, within the alternative splice variant CS-1 of fibronectin. For example, integrins like α5β1, αvβ3, and αvβ5 are implicated in the migration of cancer cells, such as in breast cancer (Felding-Habermann et al., 2001; Mierke et al., 2011) and, therefore, have been identified as biomarkers for cancer progression. It was found that integrins α2β1 and α3β1, unlike α6 integrins, are involved in the migration of melanoma cells on type IV collagen and laminin (Klein et al., 1991; Melchiori et al., 1995). Integrins can act in a synergistic manner in the development of adhesion complexes, for instance as receptors such as syndecan (Mostafavi-Pour et al., 2003). For example, syndecan-4 cooperates with different integrins, α5β1 and αvβ3, whereas syndecan-1 activates integrins in an indirect manner via interactions with growth factor receptors, such as the insulin-like growth factor receptor (Woods et al., 2000; Beauvais and Rapraeger, 2010; Karimi et al., 2018). These interactions influence important cellular processes like movement, invasion, interference between two adjacent cell types, and cell survival. For instance, the stiffness of the ECM can affect how cancer cells migrate and propagate. Cells can perceive alterations in the stiffness of the ECM and react to them, a process referred to as durotaxis (Sunyer et al., 2012; 2016). In terms of cellular forces and motility, cancer cells can exert and react to mechanical forces such as tension, compression, and shear. The cytoskeleton, a framework of actin filaments, intermediate filaments, and microtubules inside the cell, serves a crucial purpose in generating force and cell motility. Modifications in the architecture and dynamics of the cytoskeleton can influence the shape, migration, and invasion of cancer cells. The TME, comprising the ECM, blood vessels, and other cells encircling the primary tumor, is critical for moving successfully through the metastatic cascade. The physical features of the TME, such as stiffness and fluid pressure, can impact how cancer cells function (Figure 7). For instance, increased IFP can promote the spread of cancer cells.

As cancer cells spread, they may undergo EMT (partial or hybrid), a mechanism by which cancer cells acquire the characteristics of mesenchymal cells, thereby gaining the ability to migrate and invade. Mechanical forces can impact EMT, and the modified mechanical characteristics of cancer cells passing through EMT can compromise their capacity for metastasis. Another mechanism employed by cancer cells is the jamming-to-unjammimg transition, which is distinct from EMT (Mierke, 2025a). During the jamming-to-unjammimg transition, solid-like stationary cancer cells become fluid-like and invasive. Cancer cells can perceive mechanical signals from their environment, a process known as mechanosensing, and translate these signals into biochemical reactions, a process known as mechanotransduction.

Abnormal mechanosensing in cancer cells can promote or inhibit cancer advancement and metastasis. Fundamentally, cancer cells are not merely passive observers in their microenvironment; they actively perceive mechanical signals and react to them, which can promote or impede their metastasis. Knowledge of these mechanobiological mechanisms is decisive for the design of new therapeutic approaches to combat and prevent cancer metastasis. In addition to the mechanical properties of the TME, metastasis may depend on whether the endothelial cells carry certain receptors on their surface that promote transmigration and whether ECM-migrating tumor cells are flanked by other cells, such as immune cells or cancer-associated fibroblasts (CAFs). The exact mechanism has not yet been extensively researched, and a systematic analysis is needed here. Utilizing a 3D co-culture model for angiotropism and pericytic mimicry (Lugassy and Barnhill, 2007), it was additionally demonstrated that the engagement of melanoma cells with endothelial cells induced differentially expressed genes linked to cancer advancement (Bentolila et al., 2016). These genes included 10 that were also implicated in inflammation, one of which was SERPIN B2. Notably, inflammation can promote angiotropism, pericytic mimicry, and metastasis in a genetically modified mouse model for melanoma (Lugassy and Barnhill, 2007), once again highlighting the relevance of angiotropism and pericytic mimicry for the progression of melanoma and the development of metastases.

In summary, the extraluminal metastasis route is an alternative for malignant cancer progression. Nevertheless, it is still not clear whether primary cancers can alter their metastasis route by switching from luminal to extraluminal or the possibly interfacial metastasis route. When it is possible to switch metastasis routes from luminal to extraluminal, this may be driven by altering the mechanical characteristics of cancer cells, such as stiffness. In the luminal route, softer cancer cells may better withstand mechanical stresses and, hence, it can be hypothesized that softer cancer cells are better suited to utilize the luminal metastasis route. In the extraluminal route, stiffer cancer cells may adhere better to the basal lamina and the endothelial cells of the vessels. Thus, it can be hypothesized that the cancer cells of the extraluminal metastasis route need to be stiffer. In the intravascular metastasis route, cancer cells must “squeeze” through narrowing spaces (i.e., endothelial junctions) during their final passage through the endothelium. This requires the support of critical mechanisms, such as the active deformation of the cancer cell nucleus, the restructuring and contraction of the cytoskeleton, and the softening of the cell nucleus, which alter the mechanical properties of the cancer cell (Fischer et al., 2020). In these settings, the restructuring and contraction of the cytoskeleton supply the mechanical forces for nuclear deformation, whereas the nuclear softening, caused by the phosphorylation of lamin A/C, facilitates extensive nuclear deformation (Dahl et al., 2008; Davidson et al., 2015; Das et al., 2019). In some instances, enormous nuclear deformation can result in disruption of the nuclear envelope, which in turn can lead to DNA injury resulting from unregulated exchange occurring between the nucleus and the cytoplasm, along with chromatin extrusion and chromatin breakage (Denais et al., 2016; Lammerding and Wolf, 2016; Paul et al., 2017). Therefore, it can be hypothesized that the softness and deformability of cancer cells are increasingly associated with the transluminal metastasis pathway compared to the extraluminal metastasis pathway. Moreover, it can be assumed that changes in the stiffness or softness of cancer cells may cause a switch between these two routes. In addition, the discrepancy found in several studies as to whether metastatic cancer cells must be softer or stiffer, as examined in the current review (Mierke, 2025c), could be resolved. In addition to the mechanical properties of cancer cells, the influence of the basement membrane of vessels on the extraluminal metastasis pathway and signal transduction will be discussed below. All of these factors can also affect the mechanical properties of metastatic cancer cells.

## 4 Influence of the tumor microenvironment (TME) on the route of cancer metastasis

The TME can be divided into a non-cellular part that displays structural and mechanical characteristics and a cellular part that contains stromal cells and immune cells like neutrophils (see Section 5). The TME can alter the shape and functional performance of cancer cells during metastatic spread. Therefore, the structural, biochemical, and mechanical properties of the surrounding environment play a pivotal role. Both the non-cellular and cellular parts of the TME are implicated in this function. The non-cellular part of the TME constitutes the ECM, which forms a dynamic meshwork of multi-domain macromolecules that is structured in a tissue-specific organization and whose constituents cooperate to provide a structurally robust scaffold that aids in determining the mechanical characteristics of tissues (Nonnast et al., 2025). The ECM represents a reservoir of signals, provides a migration track, presents signals, contains functional fragments, and exerts biomechanical forces (Mierke, 2024d). In this context, basement membranes are specialized structures of the ECM that serve, for example, to provide optimal support for all epithelia, blood vessels, and capillaries (Pickup et al., 2014; Gopinath et al., 2022).

### 4.1 Altered mechanical properties in primary tumor tissue shape the tumor microenvironment (TME)

**FIGURE 8:**
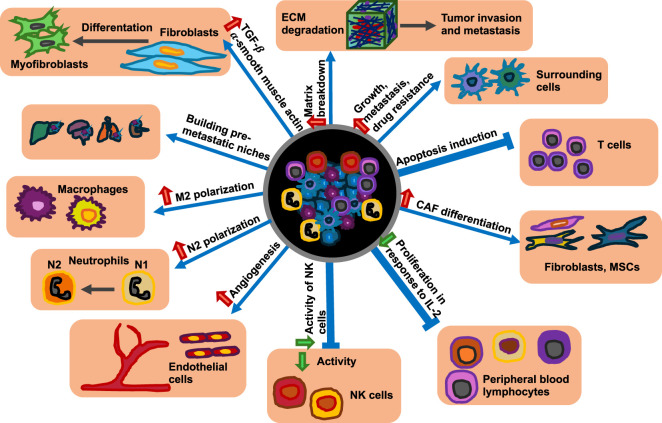
Interplay between solid primary tumors with their surrounding TME. The red arrows indicate an increase in specific functions, whereas green arrows stand for a decrease in specific functions.

There is significant interaction between the primary solid tumor and its surrounding microenvironment. In particular, the primary tumor can alter its immediate environment, giving rise to the TME (Figure 8). Moreover, it is widely accepted that altered mechanical characteristics of the primary tumor tissue change the shape of the TME. The primary solid tumor impacts the surrounding TME, which consists of disseminated cancer cells and stromal elements, including the ECM, basement membrane, vessels, immune cells such as neutrophils, and stromal cells such as fibroblasts (Figure 8).

As cancers grow, all elements change their physical characteristics and performance (De Visser et al., 2006; Pickup et al., 2014; Nyga et al., 2021). The stiffness of cancers is identified as a key physical property. Thereby, the stiffness of the TME also plays a crucial role in cancer metastasis. In most cancers, primary tumors, with a few exceptions, are generally mechanically stiffer compared to their healthy tissue of origin (Pickup et al., 2014; Streitberger et al., 2014; Nia et al., 2020; Mierke, 2025c; 2025b). Human breast tumors, for example, are five times stiffer than healthy breast tissue, and this high stiffness is linked to malignancy (Evans et al., 2010). The breast tissue of mice bearing tumors is 24 times stiffer than healthy breast tissue (Paszek et al., 2005). The stiffness of human hepatic tissue is positively correlated with the prevalence of hepatocellular carcinoma, whereby the cut-off value lies at 20 kPa (Mueller, 2010). In addition to overall stiffening, the heterogeneity of intratumoral stiffness constitutes another prominent mechanical attribute of cancerous tissue (Malandrino et al., 2018; Mierke, 2024d). Measurements using ultrasound elastography demonstrate significant spatial variation in tissue stiffness in breast and liver cancers (Mak et al., 2013). For instance, the periphery of primary tumors in human breast cancer biopsies is seven times stiffer (E = 5.51 ± 1.70 kPa) than their centers (E = 0.74 ± 0.26 kPa), while healthy breast tissue has a stiffness of 1.13–1.83 kPa (Plodinec et al., 2012). Using MRE, it has been determined that liver cancers are stiffer compared to healthy liver tissues. For instance, malignant liver tumors exhibited significantly higher mean shear stiffness than benign tumors (10.1 kPa versus 2.7 kPa), fibrotic livers (10.1 kPa versus 5.9 kPa), and healthy livers (10.1 kPa vs. 2.3 kPa) (Venkatesh et al., 2008). Fibrotic livers have a stiffness level that corresponds to that of benign and malignant cancers. A threshold limit of 5 kPa enabled a precise distinction to be made between malignant cancers, benign cancers, and healthy liver parenchyma in this MRE-based stiffness analysis (Venkatesh et al., 2008).

Moreover, metastatic lesions have been reported to be softer than cholangiocarcinoma and hepatocellular carcinoma (HCC) (Venkatesh et al., 2008). For instance, cholangiocarcinoma and HCC exhibit a pronouncedly elevated stiffness compared to fibrotic liver, benign cancers, and the normal parenchyma of the liver. Metastatic cancers showed no evidence of significantly increased stiffness compared to fibrotic liver tissue but were stiffer than all benign cancers and healthy liver parenchyma. The mean stiffness values of cholangiocarcinoma were significantly greater (16.2 kPa) than those of HCC (10.3 kPa) and metastases (7.6 kPa). HCC showed considerably higher stiffness values compared to metastatic lesions (Venkatesh et al., 2008). Nevertheless, the different stiffness values for the primary solid cancer and its metastases may be due to the properties of the metastatic niche or the shortened growth time of the metastases compared to the original primary solid cancer. In addition to stiffness, cancerous tissue also differs from normal tissue in time-dependent physical properties, such as viscoelasticity (Mierke, 2021b). For example, an *in vivo* study using magnetic resonance elastography (MRE) reveals that the fluidity of benign meningioma tissue in humans is 3.6-fold elevated compared to aggressive glioblastoma tissue. This solid-like property of glioblastomas facilitates their aggressive penetration of the neighboring tissue (Streitberger et al., 2020). Viscoelasticity has been identified at the nanoscale in single carcinoma cells and determines the overall mechanical signature of the cancer (Hadzipasic et al., 2024). From individual carcinoma cells to progressively larger carcinoma spheroids and to established cancers, there is a stepwise development of dynamic mechanical characteristics that constitute a nanorheological signature of well-established cancers. The nanorheological signature of established cancers comprises enhanced stiffness, diminished velocity-dependent stiffening, and impaired dissipation of energy (Hadzipasic et al., 2024). This development of viscoelasticity was assessed on the basis of scales and demonstrated that established cancers utilize fluid-solid interactions as the primary mechanism of mechanical energy dissipation, in contrast to fluid-independent intrinsic viscoelasticity of cancer cells (Hadzipasic et al., 2024). The energy loss mechanism observed in spheroids and established cancers correlates negatively with cell density, with this dependence being heavily influenced by an intact actin cytoskeleton. Consequently, these findings identify an emerging and targeted feature of the physical TME (Hadzipasic et al., 2024).

Over-deposition and enhanced cross-linking of the ECM, particularly of collagen, primarily raised the stiffness of the cancer tissue (Riegler et al., 2018). The TME is continuously restructured by cancer and stromal cells and supplies physicochemical information to modulate gene expression and the functions of these cells by activating a series of intra- and extracellular molecular receptors and signaling routes, such as integrin, PIEZO 1/2, and Rho/ROCK. These receptors receive extracellular biophysical impulses from the extracellular TME and pass them onto the cell nucleus (Butcher et al., 2009; Lu et al., 2012). Moreover, they subsequently convey intracellular feedback to the reorganization of the TME (Mierke, 2021a). The nature, stiffness, and organization of the ECM determine its regulatory role in cancer progression. The ECM is composed of fibrous proteins like collagen and elastin, glycoproteins like fibronectin and laminin, polysaccharides, and proteoglycans like hyaluronan (Nallanthighal et al., 2019). Elevated expression of various ECM proteins is associated with more severe disease progression in several types of cancer (Riegler et al., 2018). Abnormal expression of ECM enzymes such as matrix metalloproteinases (MMPs) or ADAM8 (Mierke, 2023), which govern ECM remodeling, is a predictor of poor prognosis (Butcher et al., 2009). As the most important structural components of the ECM, collagens contribute up to 60% of the mass of the cancer and the stiffness of the cancer tissue (Lu et al., 2012; Nallanthighal et al., 2019). The presence of high amounts of collagen promotes the development of breast cancer and invasive cancer cell phenotypes (Provenzano et al., 2008). ECM stiffness decisively influences the transformation, survival, proliferation, and motility of cancer cells. For example, increased ECM stiffness facilitates the nuclear localization of YAP, which is necessary for the normal breast cell transformation initiated through the RTK-Ras oncogene pathway (Panciera et al., 2017). Breast cancer cells exhibit elevated levels of miR-18a on stiffer ECM, which promotes cancer cell proliferation (Cassereau et al., 2015). Stiff ECM encourages the growth and invasion of breast cancer cells by creating increased cell tension. The high stiffness of the ECM strongly upregulates TWIST1, which intensifies EMT (Mierke, 2025a) and subsequently supports the metastasis of breast cancer cells (Wei et al., 2015). The EMT is distinct from the jamming-to-unjamming transition that has been reviewed in Mierke (2025a). In pancreatic ductal cancer cells, high ECM stiffness induces the signal transducer and activator of transcription 3 (STAT3) signaling route. This increases matricellular fibrosis and ductal epithelial tension and encourages the progression of the primary solid tumor through reduced TGF-β signaling and increased activation of β1 integrins (Laklai et al., 2016). Elevated ECM stiffness and increased cell contractility enhance MMP activity in pancreatic cancer cells by a factor of three to ten, thereby promoting migration, invasion, and angiogenesis (Haage et al., 2014). The three-dimensional distribution pattern of liver cancer stem cells (CSCs) matches the stiffness of the cancer tissue, meaning the outer part of the primary solid cancer is 13-fold stiffer and contains 13-fold more CSCs than the center of the primary solid cancer (Sun et al., 2021). In response to enhanced ECM stiffness, glioma cells induce the activation of Piezo1 at focal adhesion sites and amplify calcium inward flow, which activates integrin-FAK signaling and exacerbates ECM stiffening (Chen et al., 2018). Increased tissue stiffness activates the Rho/ROCK signaling route, which increases actomyosin-based cell tension and collagen attachment, resulting in improved tissue stiffness (Samuel et al., 2011). In addition to ECM stiffening, the particular stiffness of tumor tissue impacts the shape of blood vessels, how well the blood vessel barrier works, and the integrity of blood vessels (Munson and Shieh, 2014; Polacheck et al., 2014; Huang et al., 2018; Yankaskas et al., 2021). Vascular integrity appears to be an ideal property for providing support to a specific route of cancer metastasis, such as the intraluminal metastasis route or the interfacial metastasis route. Thus, the effect of vessel integrity and the “barrier” function of the endothelium is discussed below.

### 4.2 Impact of tumor microenvironment (TME) stiffness and viscoelasticity on metastatic route selection

An important question is how cancer cells decide which metastasis pathway to choose. It can be assumed that the mechanical properties of the TME, such as stiffness or viscoelasticity, play a key role. These mechanical characteristics of the TME can alter the properties of the endothelial cells like vessel integrity and barrier function lining the vessels nearby the primary solid tumors. Endothelial adhesion junctions take on a key function in modulating vessel integrity and support the barrier function of the endothelium (Dejana et al., 2008). Among the transmembrane adhesion proteins participating in cell-to-cell adherens junctions, vascular endothelial cadherin (VE-cadherin) is a pivotal cell-to-cell adhesion molecule (Dejana et al., 2008; Dejana and Vestweber, 2013). VE-cadherin attaches to p120-catenin (p120), β-catenin, and plakoglobin via its cytoplasmic domain, forms the “basic” organization of adherens junctions (Weis and Nelson, 2006; Grimsley-Myers et al., 2020). In addition, VE-cadherin connects via β-catenin and α-catenin to the F-actin cytoskeleton, ultimately forming a VE-cadherin-catenin complex. There are two possibilities for how α-catenin can interact with F-actin. The first possibility is that α-catenin directly connects the cadherin-catenin complex to F-actin. The second possibility is that other proteins, such as vinculin and α-actinin, are also able to attach to α-catenin and F-actin, resulting in additional linkages. Thereby, the VE-cadherin complex links adhesion junctions with actin-binding proteins and signaling components, affecting the actin cytoskeleton and being impacted by it (Hartsock and Nelson, 2008). Post-translational modifications of VE-cadherin are in turn decisive for the generation and controlled regulation of adherens junctions (Dejana et al., 2008; Le Guelte et al., 2011; Belvitch and Dudek, 2012; Chen et al., 2012; Jean et al., 2014). An expanding set of molecules, comprising vascular endothelial growth factor receptor 2 (VEGFR2) and vascular endothelial protein tyrosine phosphatase (VE-PTP), has been associated with VE-cadherin in addition to the traditional VE-cadherin-catenin complex (Nan et al., 2023). In the presence of VEGF, VEGFR2 engages with VE-cadherin and Src, leading to disruption of the adhesion junctions. Tyrosine phosphorylation of VE-cadherin is induced by the actions of VEGFR2 and Src kinase (Liu et al., 2020). Src and FAK have been characterized as modifiers of vascular permeability in several different studies (Thomas and Brugge, 1997; Zebda et al., 2012; Sulzmaier et al., 2014). Along with FAK, Src facilitates the tyrosine phosphorylation of VE-cadherin at Y658, Y685, and Y731, thereby compromising p120 and β-catenin engagement and reducing endothelial integrity (Potter et al., 2005; Dejana et al., 2008; Lampugnani et al., 2018). For example, FAK activity stimulated by matrix stiffness triggers Src and subsequently high concentrations of phosphorylated VE-cadherin at endothelial cell adhesion junctions (Wang W. et al., 2019). FAK is also a crucial focal adhesion protein and is implicated in the mechanical perception of cells (Schwartz, 2010; Mierke, 2013b; Bae et al., 2014; Mierke et al., 2017). FAK phosphorylation is associated with the stiffening and progression of solid tumors (Sulzmaier et al., 2014). It is noteworthy that in several types of cancer, overexpression of FAK is associated with a worse outcome for patients (Miyazaki et al., 2003; Chen et al., 2010). When cells adhere to a stiff ECM, FAK is attracted to focal adhesions and becomes phosphorylated (Miyazaki et al., 2003). FAK phosphorylation on Tyr379 generates a high-affinity binding site for Src and induces Src activation (Miyazaki et al., 2003). The FAK-Src complex acts as a signaling hub as it phosphorylates multiple constituents of focal adhesions, initiating downstream signaling pathways (Miyazaki et al., 2003; Huynh et al., 2011; Jean et al., 2014; Urbano et al., 2017). Considering the participation of FAK and Src in the integrity of the endothelial barrier, it can be postulated that stiffness-driven activation of FAK is a key player in controlling endothelial integrity. Since metastatic cancer cells produce VEGF (Yang et al., 2015), they may choose the intraluminal pathway due to the increased permeability of the endothelial cell layer of the vessels.

It has been found that matrix stiffness controls endothelial permeability via the activation of FAK (Wang W. et al., 2019). A stiffened matrix interferes with endothelial integrity and enhances permeability, whereas pharmaceutical blockade of FAK prevents the enhanced permeability caused by matrix stiffness, both *in vitro* and *ex vivo*. Moreover, stiffness-induced activation of FAK induces the translocation of Src and phosphorylation of VE-cadherin *in vitro* and *in vivo*. Inhibition of FAK hinders the translocation of Src and the phosphorylation of VE-cadherin that is caused by increased matrix stiffness. These results underscore the crucial function of FAK in modulating the matrix stiffness and thereby altering the endothelial integrity via the tyrosine phosphorylation of VE-cadherin. Increased stiffness of the ECM leads to enhanced angiogenic dispersion and increased angiogenic leakage, which undesirably favors the spread of cancer cells into the vascular system (Munson and Shieh, 2014). Thus, the intraluminal metastasis route appears to be favored by metastatic cancer cells.

Although the density and degree of collagen cross-linking govern the stiffness of the ECM, they can influence angiogenesis in opposing directions. In an *in vitro* 3D organ culture model of sprouting angiogenesis, increased matrix density reduces angiogenesis and vascular meshwork development, most likely because a stiff ECM is more challenging for endothelial cells to remodel (Shields et al., 2007). Increased collagen cross-linking supports angiogenesis in the spheroid and enhances the substrate’s stiffness (Munson and Shieh, 2014). The ECM is especially thicker and progressively more linearized in the area bordering the tumor vessels (Hockel and Vaupel, 2001). This property could help cancer cells move along these tracks toward endothelial vessels. These results suggest that the effects of increased ECM density and alignment, including cross-linking and linearization, on the stiffening and propagation of cancers are mutually exclusive. The influence of ECM stiffness on angiogenesis depends on cell-ECM adhesion. On 2D collagen-coated polyacrylamide (PA) gels, the softer ECM (200 Pa versus 10 kPa) promotes the formation of endothelial cell loops, which mimics the initiation of angiogenesis. However, these PA gels become suppressive when the collagen concentration is reduced from 100 μg/mL to 1 μg/mL. Moreover, an important observation is that elevated matrix stiffness triggers the expression of the CCN1 protein within endothelial cells that envelop blood vessels. This leads to increased levels of N-cadherin, which is an adhesion protein that enables stronger engagement of cancer cells with blood vessel walls—a crucial step for cancer cells to penetrate into the bloodstream (intravasation) and spread to remote organs. Intraluminal metastasis offers a principal route for systemic dissemination.

The impact of ECM stiffness on the progression of the tumor is, nevertheless, debatable (Mierke, 2025c). Ovarian cancer cells, for instance, tend to be more invasive when they are in a softer surrounding (McGrail et al., 2014). In contrast, higher matrix stiffness in solid tumors is linked to increased invasion and metastasis. This is attributed, at least in part, to the increase in CSC population and biomarkers (Hui et al., 2017; Pankova et al., 2019). There is emerging evidence that matrix stiffness is able to activate receptors and mechanosensory/mechanoregulatory proteins, including integrin, FAK, and YAP, thereby regulating the properties of cancer cells and CSCs via various molecular signaling routes. In a soft (90 Pa) but not stiff (1.05 kPa) ECM scaffold, it has been observed that an abrupt switch to low ECM stiffness maintains the stem cell-like nature of malignant tumor repopulating cells (TRCs) or CSCs (Liu et al., 2012; Tan et al., 2014; Tian et al., 2019; Safaei et al., 2023; Mierke, 2024d). Apart from matrix stiffness, the viscoelasticity of the ECM also plays a significant role.

Viscoelastic materials, such as collagen matrices, for example, the ECM scaffold, display a reaction that falls in between these two limits. Biological tissues are viscoelastic materials, meaning that they initially react elastically to a force and then are subject to continuous viscous deformation. As a result of this delayed viscous reaction, viscoelastic materials continuously reshape themselves over time when subjected to force, a behavior referred to as “creep.” Likewise, viscoelastic materials undergo “stress relaxation” when subjected to constant strain, i.e., the stress decreases over time. In purely elastic materials, in contrast, the stress remains unchanged over time. These responses highlight the time-dependent character of viscoelastic materials as opposed to the time-independent nature of purely elastic materials. The compliance of a magnetic platform with a high ligand tether mobility tightly controls the stemness and tumorigenicity of cancer cells (Wong et al., 2021). For instance, CD133+ liver CSCs soften local niches to preserve their stemness, improve drug resistance, and enable metastasis (Ng et al., 2021). These different reactions to ECM stiffness could be attributed to the mechanosensory dependence of certain types of cancer as well as to the presence of a heterogeneous TME and heterogeneous subpopulations of cancer cells. Collagen is a fibrous material that exhibits the characteristics of strain hardening, nonlinear elasticity, inhomogeneous mechanical characteristics, and material anisotropy (Storm et al., 2005; Sopher et al., 2018; Hayn et al., 2020). Stiffening of the ECM can be induced by collagen strain hardening even under low stress caused by cell contraction and, conversely, promotes tumor growth when the strain hardening is irreversible (Willem et al., 2002; Stylianopoulos et al., 2012). Using a finite element model, it was determined that the nonlinearity of collagen fibers, such as compression buckling and tension stiffening, is able to promote long-distance transmission of mechanical information to remote cells, for example, over even nine cell-length distances (Chang et al., 2009; Humphries et al., 2017). In addition to stiffness, ECM architecture, including fiber orientation, cross-linking, porosity, and topography, also influences the invasive behavior of cancer cells, like motility, protrusions, and the activity of MMPs within self-assembled 3D collagen scaffolds (Fraley et al., 2015; Henke et al., 2020). High collagen density leads to smaller pores in the ECM, and a moderate pore size of 5–12 μm is seen as a crucial factor in promoting glioma invasion (Yang et al., 2010). By utilizing a mutually interpenetrating mesh of hydrogels (from 30 Pa to 310 Pa), the impacts of pore size and stiffness on cancer cells could be separated from each other (Chaudhuri et al., 2014). Encapsulation of cancer cells in the pores increases their polarization, traction force, and migration velocity. Cell migration velocity is strongly related to ECM stiffness in spatially constrained ECM; nonetheless, this relationship is biphasic in unconstrained ECM (Pathak and Kumar, 2012). In breast cancer, collagen fibers that run vertically to the boundary of the primary tumor facilitate invasion and metastasis (Provenzano et al., 2008). This is due to a high degree of collagen cross-linking combined with enhanced ECM stiffness, which eases the invasion of cancer cells by strengthening integrin-regulated FAK-Src signal transduction (Levental et al., 2009).

What is the effect of the viscoelasticity of the TME on the choice of the metastatic route? When examining the mechanical properties of cancer cells, it was found that cancer cells themselves tend to be softer and more deformable compared to normal cells, which presumably supports their capacity to move through dense tissues and blood/lymph vessels as they metastasize. Nonetheless, it remains unclear whether the viscoelasticity of the surrounding ECM can impact the mechanical properties of cancer cells and, consequently, their choice of metastatic route. There is interesting knowledge about the capacity of cancer cells to alter the mechanical characteristics of their surrounding ECM (Huang et al., 2025). Specifically, the tumor-associated ECM viscoelasticity alteration is the consequence of coordinated ECM component control. Collagen fibers, which are the primary structural elements of the ECM, determine its viscoelastic characteristics by their density, cross-linking, and three-dimensional positioning (Courbot and Elosegui-Artola, 2025). For instance, in type 2 diabetes-associated nonalcoholic steatohepatitis (NASH) models, advanced glycation end products (AGEs) markedly increase viscous dissipation and accelerate stress relaxation by reducing collagen fiber length and decreasing network connectivity (Fan et al., 2024). Thus, shorter fiber length, larger heterogeneity, and decreased interconnectivity enhance the viscoelasticity of the AGE-bundled collagen matrix. Moreover, hypoxia triggered by enhanced ECM stiffness induces the activation of the reactive oxygen species (ROS) signaling pathway, thereby increasing MMP-1 expression, enhancing collagen breakdown, and favoring the generation of cell clusters, which collectively serve to control ECM viscoelasticity (Blatchley et al., 2019). Hyaluronic acid, a key element of the ECM, displays bidirectional regulatory properties on viscoelastic characteristics that are dependent on its molecular weight. High-molecular-weight hyaluronic acid improves matrix viscosity by increasing hydration interactions, whereas low-molecular-weight hyaluronic acid enhances matrix plasticity through increasing network fluidity (Lou et al., 2018). Specifically, the viscoelasticity of the ECM exhibits considerable heterogeneity between various cancer types and even within the same tumor. Rubiano and coworkers quantified the viscoelastic characteristics of resected human pancreatic tissue utilizing a specially designed cantilever-based indentation device (Rubiano et al., 2018). They extracted crucial variables, such as the steady-state modulus (SSM), viscosity, and characteristic relaxation time, after fitting the stress relaxation curves with the Standard Linear Solid (SLS) model. Their study showed that pancreatic ductal adenocarcinoma (PDAC) cancers displayed increased SSM values and viscosity, whereas tissue with chronic pancreatitis showed markedly reduced viscosity, although stiffness was elevated (Rubiano et al., 2018). Onwudiwe and coworkers compared the viscoelastic behavior of the cell nucleus and cytoplasm of the human GBM cell line U87 with that of normal immortalized human astrocytes (IHAs), the presumed cells of origin of GBM (Onwudiwe et al., 2024). Specifically, they simulated successfully physiological shear stress within the brain in a 2D setting using a microfluidic device and detected no relevant variation in viscoelastic properties (stiffness, viscosity, and relaxation time) of IHA and GBM cells (Onwudiwe et al., 2024). The remarkable viscoelasticity and spatial heterogeneity of tissues highlight the enormous importance of viscoelasticity as a tissue-specific mechanical hallmark and therapeutic objective. The mechanical characteristics of tissues are predominantly determined by the ECM, which can be reproduced *in vitro* utilizing hydrogels, as they can replicate the architecture and functionality of natural ECM scaffolds (Hayn et al., 2020). In addition, hydrogels are generally viscoelastic, and their viscoelasticity can be conveniently tailored by altering material variables such as crosslinking type and molecular weight. They thus provide an ideal model for exploring the relationship between the viscoelasticity of the ECM scaffold and the advancement of cancer. Consequently, insights into the interplay between ECM viscoelasticity and the malignant progression of cancer, gained from bioengineered cancer models, will be critical for the identification of specific biomechanical markers (Mierke, 2024a). This can contribute to more accurate and earlier diagnosis and predictions, along with more powerful treatment choices. Ultimately, it can only be speculated how cancer cells are influenced by the mechanical properties of the TME, such as stiffness and viscoelasticity, in their choice of metastasis route. For example, it is conceivable that stiffer cancer cells use the extravascular route, while softer ones use the intraluminal route. It is also plausible that cancer cells with greater viscoelasticity choose the intraluminal route of metastasis, while cancer cells with lower viscoelasticity choose the extraluminal route. Nevertheless, another scenario is also possible when ECM stiffening arises. A high collagen content and its cross-linking (by the enzyme LOX) increase tissue tension. This activates signaling pathways, such as FAK/Src, which drive cancer cells to invade and enter the bloodstream. High solid stress, resulting from the growth of the primary tumor, can cause the lymph vessels to collapse, which paradoxically temporarily “diverts” metastatic spread to the hematogenous route until new lymph vessels are formed. Thus, higher stiffness directly promotes local, tissue-based invasion via an extraluminal metastasis route. While vascular invasion (intravasation) tends to be influenced by fluid pressure (interstitial pressure), the “breaking through” of tissue boundaries is primarily a result of altered tissue viscoelasticity and stiffness. High pressure in combination with increased fluid flow (interstitial fluid flow, IFF) at the margin of the primary tumor can promote the migration of cancer cells and ease their intravasation into blood vessels (Rice et al., 2017).

High ECM stiffness serves as a critical biomechanical stimulus via PIEZO1-regulated calcium inflow, which triggers epithelial-mesenchymal transition (EMT) and facilitates the migration, invasion, and metastatic spread of cancer cells (Lopez-Cavestany et al., 2023). Enhanced stiffness initiates signaling pathway activation (such as TWIST1, Snail, and YAP/TAZ), which represses epithelial markers, such as E-cadherin, and enhances mesenchymal markers, such as vimentin. EMT not only softens cancer cells, but also enhances the release of MMPs, which helps build migration tracks for cancer cells (van Helvert et al., 2018). Invadopodia, which consist of actin-based protrusions of the plasma membrane, exhibit enzymatic activity for breaking down the ECM scaffold (Eddy et al., 2017). In a stiffer ECM environment, cancer cells encourage their infiltration into the ECM by raising the amount of invadopodia, thus easing invasion and migration (Artym et al., 2015). Consequently, plasticity changes cause cancer cells to soften during cancer migration, and the greater number of invadopodia encourages ECM breakdown, thereby improving their capacity for invasion and migration. Finally, more experiments are required to substantiate these hypotheses. Specifically, it needs to be explored whether and how cancer cells can switch between different metastasis routes due to altered mechanical microenvironmental cues.

### 4.3 Adaptation to stress and external forces by cancer cells

**FIGURE 9:**
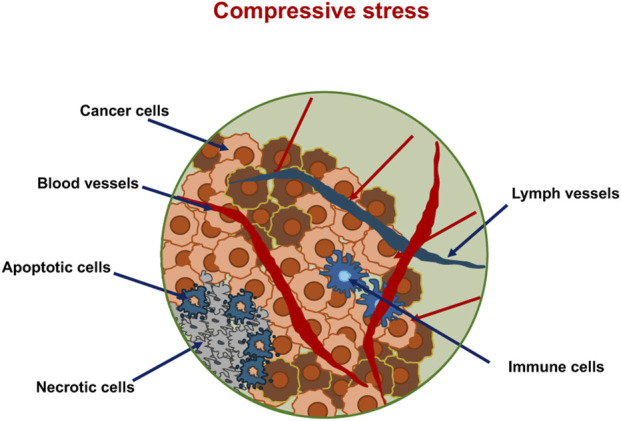
Compressive stress acts on the primary tumor, which includes cancer cells, blood and lymph vessels, and immune cells.

While undergoing all these consecutive steps of metastasis, cancer cells are exposed to different and varying external mechanical stresses that threaten their survival and spread and necessitate specific mechanical characteristics. In the initial phases of tumor growth, cells are subjected to growing compressive stresses (Figure 9), each of which is attributable to inequalities in the inflow and outflow of fluids into the interstitial space (Mohammadi and Sahai, 2018) and the microenvironment that restrains the growing tumor volume (Stylianopoulos et al., 2012; Jain et al., 2014). The increase in compressive stress is attributable to the combined effects of tumor volume expansion and TME stiffening as a result of stroma restructuring, which develops in a variety of tumor types, including breast and pancreatic cancer. In addition, the huge volume of cancerous tissue can also compress blood vessels and lymph vessels, along with the ECM, leading to extravasation of tissue fluid and elevated IFP (Nia et al., 2020; Purkayastha et al., 2021).

Consequently, the compressive stress will continue to rise (Tan et al., 2024). Stroma remodeling is primarily directed by stromal cells such as CAFs and primarily comprises topographical disruption and stiffening of the ECM. It is well-established that it promotes invasion and metastasis (Goetz et al., 2011; Pickup et al., 2014; Clark and Vignjevic, 2015), especially by collagen cross-linking, integrin clustering (Paszek et al., 2005; Levental et al., 2009) and, above all, by the inducement of paths that are generated through radially oriented fibers (Provenzano et al., 2006). These tracks can be utilized for collective cancer cell migration with the assistance of guiding fibroblasts (Gaggioli et al., 2007). Nevertheless, the impact of rising compressive stress due to the restructuring of the stroma involves a decline in tumor cell size, a lack of proliferation, and a stalling of the cell cycle, which collectively impede the growth of the tumor, as demonstrated by *in vitro* investigations (Delarue et al., 2014; Taubenberger et al., 2019). Due to tumor volume expansion, elevated compressive stress, and ECM stiffening, cancerous tissue is typically stiffer than healthy tissue. Clinically, it has been proven that malignant tumors are considerably stiffer compared to their benign equivalents (Lorenzen et al., 2002; Venkatesh et al., 2008). A confining primary tumor milieu not only promotes compressive stress but also encourages collective cancer cell invasion rather than single-cell invasion (Haeger et al., 2014). These effects could promote the intravasation of invasive, rapidly developing CTC clusters, whose metastatic capacity is 50 times greater than that of individual CTCs (Aceto et al., 2014). Intriguingly, cancer cell clusters have been observed to express keratin-14 in certain models (Cheung et al., 2016), which may be linked to increased stiffness (Seltmann et al., 2013) when compared to cancer cells that have experienced EMT and instead exhibit high vimentin expression. The effects of cancer cell clustering on the mechanical characteristics and mechanical fitness of cancer cells are still largely unclear and are also briefly discussed in this overview.

What about rupturing forces on metastatic cancer cells? Cancer cells are exposed to mechanical stress in the vicinity of the primary tumor and, in particular, following intravasation when they move through the circulation and undergo the intravascular phases of the metastasis cascade (Follain et al., 2020). As soon as they enter blood circulation (or lymphatic system), cancer cells are subjected to completely dissimilar mechanical stresses (Figure 7) caused by hemodynamic forces and narrowing blood vessels. CTCs must cope with these stresses to successfully finish the metastasis cascade (Follain et al., 2020). For instance, it has been demonstrated that high fluid shear forces (> 1 Pa) can cause cell cycle arrest (Chang et al., 2008) or even kill cancer cells while they are circulating (Regmi et al., 2017). Ultimately, a limited number of CTCs may evade the impact of fluid shear stress and have the chance to arrest and attach to the endothelium. It is noteworthy that clusters of CTCs have also been demonstrated to pass through capillary-sized vessels, which could provide additional protective benefits (Au et al., 2016).

**FIGURE 10:**
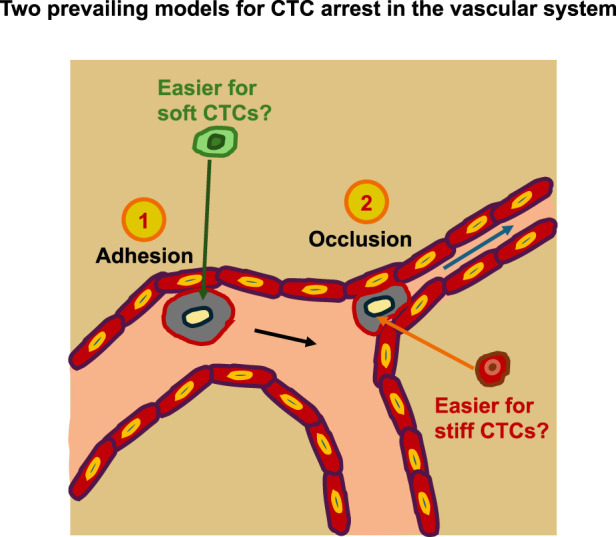
There are two prevailing models of the arrest of CTCs in the vasculature. In the first model, CTCs adhere to the vessel wall within the lumen and remain in a locked state. It is assumed that softer CTCs (green) adhere more easily to the endothelium. In the second model, CTCs get stuck in the narrow vessels, leading to their occlusion, and remain in a locked state. It can be assumed that it is easier for stiffer CTCs (red) to occlude small vessels such as capillaries.

There are two predominant models for the arrest of CTCs in the bloodstream: they could either become arrested via active adhesion to the vascular walls (Follain et al., 2018; Osmani et al., 2019) or via occlusion when the vessel is topographically disorganized and/or has only a small diameter (Kienast et al., 2010; Wirtz et al., 2011; Headley et al., 2016; Entenberg et al., 2018; Paul et al., 2019) (Figure 10). The latter condition frequently arises in microcapillaries, where CTCs are compressed as they must pass through narrow constrictions to arrive at extravasation sites close to their target organs. Ultimately, successful macrometastatic growth is triggered through the extravasation of arrested CTCs into perivascular areas. In this step, metastatic cells must overcome hurdles such as the vascular wall or the blood-brain barrier (in the case of brain metastases) in order to either become dormant or multiply and form life-threatening metastases.

All mechanical stresses to which cancer cells are exposed during these successive phases of the metastasis cascade clearly affect their internal mechanical condition. Fine-tuning their mechanical characteristics could enable cancer cells to adjust to the diverse mechanical stresses that pose a threat to the metastatic process at each stage. Specifically, their own adaptable mechanical phenotype could be crucial for handling the stresses being discussed. While this is probable, there appears to be limited experimental evidence that cancer cells alter their mechanical signature when they acquire specific mechanical or other signals as they advance down the metastatic cascade. Predominant concepts are debated, and speculation arises as to how the mechanical characteristics of cancer cells might develop alongside the cascade. While not the central focus of this review, chemical and biochemical cues such as pH, temperature, cytokines, adhesion receptors, and oxidative stress may also be implicated in the mechanical adaptation of cancer cells (Sunyer et al., 2009; Sun et al., 2014; Scholz, 2018). The mechanical profiles of cancer cells throughout their metastatic progression are briefly addressed, thereby supplementing, actualizing, and expanding upon previous reviews on this theme (Suresh, 2007; Kumar and Weaver, 2009; Wirtz et al., 2011; Mierke, 2014). Moreover, it is explained how these mechanical profiles are triggered, guided, and disrupted. In addition, speculation is offered as to how their deformability abilities could support cancer metastasis, and emphasis is placed on why these factors will be important research approaches in the field going forward. Ultimately, the idea is that the image of a clearly defined static mechanical behavior of a cancer cell does not align with the diverse mechanical characteristics required for successful metastasis. Alternatively, it could be the capacity to dynamically adjust to these demands that is the mechanical hallmark of a thriving metastatic cancer cell. In addition to the ECM properties of the TME, the basal lamina of vessels seems to be crucial for cancer cells on their metastatic journey.

### 4.4 Role of the composition and structure of the basal lamina in the extraluminal metastatic route

**FIGURE 11:**
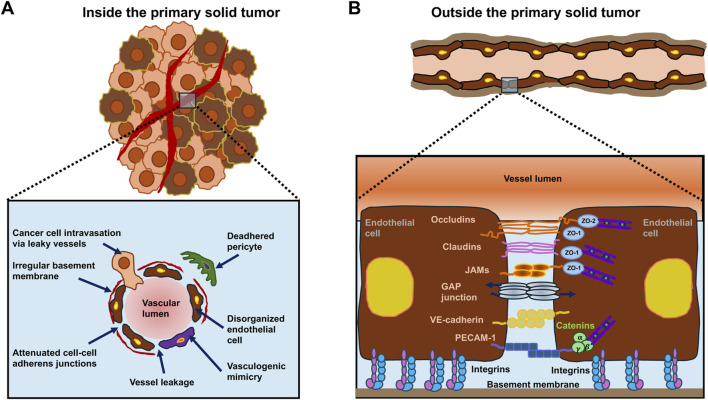
Different structural features and important molecular constituents of blood vessels in the context of their positioning. **(A)** Structure of tumor-associated vessels with unorganized endothelial layers, irregular basement membranes, dissociated pericytes, and vasculogenic mimicry, which is a mechanism whereby aggressive cancer cells create blood vessel-like patterns without or with the participation of endothelial cells, allowing tumors to absorb nutrients and oxygen regardless of normal blood vessels. Dislodged cell-cell adhesion complexes facilitate vascular permeability and intravasation of cancer cells. **(B)** Membrane receptors crucial for maintaining healthy vascular integrity include tight junction proteins (occludin, claudin, and JAMs), adhesion molecules (PECAM-1 and VE-cadherin), gap junctions, and catenins.

The basement membrane (or synonymously basal lamina) is a thin (20 nm–10 µm thick) and porous (10 nm–112 nm pore size) cross-linked ECM layer composed primarily of collagen IV and laminin. It divides cancer tissue from the surrounding normal/stromal tissue and forms the exterior border of blood vessels (Paulson, 1992). Unlike healthy basement membranes, cancer basement membranes are thinner, less organized, and contain reduced levels of collagen IV and laminin (Zhang et al., 2002; Berg et al., 2012). The compromised integrity of the basement membrane constitutes a significant characteristic of numerous types of cancer (Albrechtsen et al., 1981). When cancer cells begin to metastasize, they are likely to come into contact with endothelial vessels both inside and outside the primary solid cancer. The endothelial vessels of the tumor are vulnerable in terms of their integrity and may no longer be able to support pericytes, which then presumably detach (Figure 11A). Outside the tumor, the vessels form a stable entity that cancer cells must cross before they can spread intraluminally (Figure 11B).

The constitution of the basement membrane has a considerable influence on metastasis, acting to promote or impede it, mainly through the adjustment of its own structural integrity and stiffness, as well as the capacity of cancer cells to engage with it. This raises the question of how the basal lamina influences the extraluminal metastatic route. Angiotropic tumors are characterized as tumors that exhibit pericellular localization and are directly connected to the basal lamina of endothelial cells. From an ultrastructural perspective, the basal lamina constitutes an amorphous matrix. Laminin is the most important non-collagenous constituent of ECM proteins, implicated in cell migration, adhesion, and differentiation. Laminins are heterotrimeric glycoproteins consisting of various combinations of alpha (with five genetic variants), beta (with four genetic variants), and gamma (with three genetic variants) chains. In angiotropic melanomas, the microvessels of the cancer exhibited a minimal focal vascular positivity for α3, β3, and γ2 laminin, whereas the vessels of the cancers were marked with a positivity for β2 laminin. Cancer cells in this setting disseminate on the abluminal surface of small vessels, which are rich in β2-laminin (Lugassy et al., 1999). Changes to the basal lamina caused by neutrophil elastase break down laminin-332 (α3, β3, and γ2) and increase the potential for melanoma cells to spread (Landsberg et al., 2016). Correspondingly, *in vitro* and *in vivo* studies clearly demonstrated that the C16 peptide (KAFDITYVRLKF), which is derived from the laminin-γ1 chain, increases the angiotropic extravascular migration and lung metastasis of melanoma cells (Kuratomi et al., 2002). LAMC2, LAMA4, and ITGB3 expression is elevated in vascularized angiotropic melanoma regions compared to the avascular regions of the same tumor (Lugassy et al., 2009). In addition, the expression of laminin receptors is an adverse prognostic determinant in melanoma. CD36, which is a membrane glycoprotein implicated in angiogenesis, promotes vascular mimicry in human melanoma cancer cells by operating through the α3 integrin-laminin crosstalk (Martini et al., 2021). An *in silico* analysis of CD36 expression in the melanoma cohort of the Cancer Genome Atlas indicates that melanoma sufferers expressing high levels of CD36 tend to experience worse clinical prognosis (Martini et al., 2021).

Changes in the composition of the basement membrane can result in increased stiffness, which hampers cell penetration, or the formation of a more porous or disrupted barrier, both of which favor cancer cell transmigration. For instance, elevated stiffness is accomplished through enhanced accumulation of constituents such as collagen and fibronectin, both of which may, in turn, encourage the invasion by activating CAFs that restructure the tissue. In contrast, the breakdown of other constituents can lead to the deterioration of the basement membrane. For instance, the degradation of specific basement membrane proteins, like heparan sulfate proteoglycans, can harm the basement membrane, thereby permitting cancer cells to penetrate more easily. In addition, alterations in the composition of the basement membrane can increase the permeability of blood vessels, enabling cancer cells to more readily penetrate and leave the circulation. Modifications in laminins, such as in specific laminin variants, can foster the growth of tumors and increase the malignant phenotype. Thus, the question can be raised of how the modified composition of the basement membrane impairs cancer metastasis. First, the structural integrity is compromised. For instance, a healthy, undamaged basement membrane functions as a physical barrier that blocks cancer cells from infiltrating the adjacent tissue. Second, there are the growth-promoting activities of certain constituents. For instance, some constituents of the basement membrane, such as laminin, are able to stimulate the growth of tumors and consequently the malignant phenotype by increasing cancer cell growth and the activity of proteases. What factors can impact the composition of the basement membrane and, subsequently, cancer metastasis? First, cancer cells can modify the composition of the basement membrane by secreting enzymes and other substances, such as ECM proteins, or by releasing exosomes (Mierke, 2024d), and stromal cells can also participate in the reorganization and stiffening of the basement membrane. Second, genetic mutations and epigenetic alterations are capable of enhancing a cell’s propensity for metastasis by changing its interfacial behavior with the basement membrane. Third, inflammatory cues such as TNF-α can alter the composition of the basement membrane, thereby predisposing it to cancer cell invasion. In the following, the effect of signaling molecules on the extraluminal route of metastasis is discussed.

### 4.5 How do signaling molecules affect the extraluminal metastatic route?

Another major question is what kind of molecules are involved in the extraluminal metastatic route. Moreover, what regulates the extraluminal metastatic route of cancer metastasis? It has been resolved by Lugassy and coworkers for melanoma patients experiencing this alternative route of metastasis using gene expression analysis of 26 out of 66 patients with metastatic spread (Lugassy et al., 2011). Tissue factor pathway inhibitor 2 (TFPI-2) has been characterized as a Kunitz-type serine protease inhibitor (Chand et al., 2005). TFPI2 enhances perivascular migration in a melanoma angiotropism model (Mo et al., 2021). To observe the reciprocal interactions between human melanoma cells and the vascular system *in vivo*, a murine co-xenotransplant model was utilized, in which highly and weakly invasive melanoma cells were injected subcutaneously together. Through the blocking of several proteinases, among them plasmin, chymotrypsin, trypsin, elastase, and metalloproteinases (Rao et al., 1995; 1999; Herman et al., 2001), TFPI2 is able to suppress cancer cell invasion and metastasis by controlling the breakdown of the ECM. Downregulation of TFPI2 has been observed in different cancer types and is linked to the progression of malignant cancers (Wojtukiewicz et al., 2024). Also, methylation of TFPI2 is able to forecast the outcome and metastasis of malignant cancers (Izumi et al., 2000; Nigro et al., 2013; Sun et al., 2016; Zhao et al., 2020). Notably, downregulation of TFPI2 reduces the perivascular migratory activity of highly aggressive melanoma cells. It was hypothesized that TFPI2 might impede the angiotropic phenotype of melanoma. Because of these findings, TFPI2 is considered to act as a tumor suppressor gene. However, TFPI2 has also been shown to have pro-invasive and pro-metastatic properties, meaning that it promotes the growth of cancer cells in the lung. This is a paradoxical effect for a protein that is known to inhibit the growth of cancer cells. Moreover, it has been demonstrated that TFPI2 also facilitates the migration of cancer cells, encourages the tight adhesion of endothelial cells, and increases the creation of vasculogenic meshworks through aggressive melanoma cells (Iino et al., 1998; Neaud et al., 2000; Ruf et al., 2003). It has been proposed that TFPI2 exerts an anti-angiogenic effect by directly blocking action on endothelial cells (Xu et al., 2006). The mechanism behind the various effects of TFPI2 is still poorly understood. Several *in vivo* and *in vitro* models have been employed to investigate the development and mechanisms of angiotropism in various types of cancer. It therefore stands to reason that an innovative and efficient angiotropic model for melanoma has been identified (Mo et al., 2021). Based on this model system, differentially expressed genes and signal transduction routes/biological processes linked to melanoma angiotropism were assessed. In addition, it has been established that TFPI2 facilitates the angiotropic migration of melanoma cells and that increased TFPI2 expression levels relate to a lower or higher survival rate in UM and CM patients, respectively, which suggests different actions in different contexts. These findings could indicate distinct, possibly contradictory functions of TFPI2 for the survival rates of UM and CM patients (Mo et al., 2021). Nevertheless, since TFPI2 is upregulated by VEGF produced by metastatic cancer cells (Yang et al., 2015), there is a discrepancy due to the choice of metastasis pathway, as VEGF appears to increase vascular endothelial permeability and thereby promote the intraluminal pathway, while TFPI2 appears to support the extraluminal pathway. It can also be speculated that these are discrete concentrations that can dynamically support either of the two pathways; for example, cancer cells may change their metastasis pathway in response to external signals. Inflammatory factors, such as lipopolysaccharide and TNF-α, gently increase the TFPI-2 gene expression level within the endothelium (Iino et al., 1998). Likewise, IL-1β induces an above tenfold increase in TFPI-2 within the endothelium within the acute stage of the inflammatory process (Xu et al., 2006). This leads to the hypothesis that inflammation in the TME could contribute to the selection of the extraluminal metastasis route. Ultimately, further experimental work is needed to determine the exact mechanisms.

### 4.6 How do mechanical cues from the basal lamina affect the extraluminal metastatic route?

Mechanical signals from the basal lamina considerably impact the extraluminal route of metastasis by functioning both as a physical barrier and as a mechanical signal transmitter. However, the endothelial cell layer has been shown not to represent a physical barrier; rather, it increases the migration and invasion of specific cancer cells, such as human MDA-MB-231 breast cancer cells (Mierke et al., 2011). Alterations in the stiffness, tension, and constitution of the basement membrane during the development of a tumor directly influence the capacity of a cancer cell to infiltrate the underlying tissue and form metastases. All characteristics of a cancerous basement membrane, such as weakened integrity, reduced thickness, increased discontinuity, and reduced cross-linking (Zhang et al., 2002; Berg et al., 2012), indicate a potentially less rigid basement membrane. However, this is not the case. Instead, the basement membrane is found to be a lot stiffer in numerous cancerous tissues, such as about 10 kPa for colon, skin, and breast cancer, compared to normal tissues, such as about 3 kPa for breast glands and about 0.12 kPa for prostate glands (Swaminathan et al., 2011; Rathje et al., 2014; Dai and Siemann, 2019; Han et al., 2020; Matthews et al., 2020). Crucial processes take place at the basement membrane, such as whether its barrier function prevents the infiltration of cancer cells or whether the invasion of cancer cells prevails. The basement membrane is usually a dense sheet of ECM that physically prevents the passage of cells like cancer cells. Cancer cells usually have to break down this obstacle with the aid of proteases, such as MMPs, or exert adequate physical force to break through it. There is also a relation between matrix stiffness and cancer cell invasiveness. For instance, increased stiffness in the surrounding matrix and the basement membrane itself commonly promotes the aggressiveness and invasiveness of cancer cells. The stiffer matrix enables cancer cells to create larger traction forces, which eases migration into the neighboring stroma. Specifically, cancer cells can exert physical forces together while stretching and thinning the basement membrane as a balloon, thereby weakening it and enabling the cancer cells to break free. This implies that a physical, protease-autonomous invasion mechanism may interact with conventional protease-dependent mechanisms. Cells are able to penetrate the basement membrane through both proteases and mechanical forces. Increased stiffness of the collagen I matrix can elevate MMP activity at sites where cancer cells protrude to break down the ECM and initiate invasion (Yang et al., 2010; Malandrino et al., 2018; Cox, 2021). Nevertheless, it is unclear whether this also holds true for the basement membrane, which mainly consists of laminin and collagen IV. During the process of angiogenesis, cancer cells break down the prevalent basement membrane through proteases and facilitate the initial laminin polymerization via surface proteins and the basement membrane formation (Willem et al., 2002). This restructuring procedure can lead to untight circulatory vessels inside the primary solid cancer and thus promote the emergence of metastases (Kalluri, 2003; Chang et al., 2009). In a force-driven fashion, CAFs produce a mechanical force to enlarge the existing void in the basement membrane to 6.2 ± 1.7 µm in diameter and soften the basement membrane, which eases invasion by cancer cells in a way that is independent of MMP (Glentis et al., 2017). Cancer cells are able to perceive mechanical tension *in vitro* and possibly elevated tension of the basement membrane *in vivo*. An important but still unresolved issue is how tension in the basement membrane, which is physically linked to the growth of the tumor, leads to the attenuation of the basement membrane and thereby affects downstream signal transduction in the cancer cells adjoining the basement membrane. Moreover, mechanotransduction takes on a certain function. Cancer cells detect these mechanical alterations via transmembrane receptors, notably integrins and PIEZO1/2 receptors. This mechanosensing actuates intracellular signal transduction routes, such as RhoA, PI3K/AKT, Src/ERK, and YAP, that rearrange the cytoskeleton of the cells, enhance cell contractility, and improve migratory and invasive abilities. In addition, there are several orientation aids for cancer cells. For example, the mechanical and structural characteristics of the matrix, such as the orientation and density of fibers, can provide directional cues to migrating cells and guide them along the path of least resistance or toward blood vessels for intravasation. Caused by changes in the constitution of the basal lamina components, such as the ratio of netrin-4 (NET4) to laminin, they are able to “soften” the matrix, which may actually reduce the invasive capability of cancer cells in certain situations, suggesting a complex relationship between different physical and biochemical elements. NET4 incorporates an N-terminal domain that tethers laminin γ1 to the molecular interface where laminin β1 would normally bind, thereby preventing the formation of heterotrimeric laminin polymer nodules composed of α1, β1, and γ1 subunits and consequently their polymerization into an extracellular lattice network (Kulczyk et al., 2025). The four-fold higher affinity of the NET4-laminin-γ1 binding is due to the larger entombed surface area compared to that produced by β1-and γ1-laminins and larger electrostatic surface complementarity. Additionally, NET4 is able to disassemble already existing laminin sheets (Kulczyk et al., 2025). Consequently, the mechanical characteristics of the basement membrane are key determinants of cancer cell performance and actively impact how they overcome physical obstacles to trigger the extraluminal phase of metastasis.

How are mechanical signals able to regulate the general choice of metastasis route? The reduction of cell stiffness through the pharmacologic blockage of myosin II in human ovarian cancer cell lines, like OVCA429 and IGROV cells, enhances their invasiveness. In contrast, increasing cell stiffness through the restoration of the expression of the metastasis suppressor TβRIII/betaglycan reduces their invasiveness. Nevertheless, the underlying mechanisms are not yet fully elucidated. Infiltrating cancer cells must penetrate the basement membranes in a physical manner as they metastasize (Huang et al., 2019). Consequently, the mechanical characteristics and constitution of the basement membrane have a decisive influence on the invasion of cancer cells (Kalluri, 2003; Reuten et al., 2021). For instance, NET4-facilitated softening of the basement membrane (25 kPa compared to 50 kPa) reduces the invasion of mouse breast cancer cells, although NET4 leads to elevated pore sizes (Reuten et al., 2021). Elevated stiffness of substrates resembling the basement membrane diminishes the clustering of integrin α6β4 and uncovers its multiple locations for phosphorylation through receptor tyrosine kinase (RTK), thereby activating PI3K and Rac1 signal transduction, which leads to the induction of a malignant phenotype (Chaudhuri et al., 2014). Nevertheless, another report indicates that reducing basement membrane stiffness via targeted manipulation of Col4a1 increases cancer cell invasion in mice (Fiore et al., 2020). The stiffness and constitution of the basement membrane cooperate to control the invasiveness of cancer cells (Chaudhuri et al., 2014). A stiff basement membrane makes it easier for MCF-10A cells to invade, whereas increased laminin density causes the basement membrane to become stiffer and prevents normal breast cell clusters from invading, indicating the complex functions of basement membrane constitution and stiffness in cancer cell invasion (Pasco et al., 2005). It is therefore uncertain whether the targeted manipulation of Col4a1 influences the invasion of cancer cells by affecting the stiffness or constitution of the basement membrane. In addition to the elastic modulus, the plasticity of the basement membrane, which is the ability of a material to maintain a deformation permanently, also impacts the performance of the cancer cells. For instance, covalent cross-linking of a reconstituted basement membrane with tissue transglutaminase decreases the plasticity of the basement membrane but preserves a comparable modulus of elasticity. The basement membrane exhibits slow tension relaxation, which counteracts the spread and protrusion development of breast cancer cells (Wisdom et al., 2020). The following section discusses the cellular part of the TME with an emphasis on neutrophilic cells.

## 5 Role of neutrophilic cells in intra- and extraluminal metastasis

Many immune cells play a role in cancer metastasis, as outlined in (Mierke, 2024d), and potentially especially in the selection of the metastasis pathway. The interplay between CTCs and neutrophilic cells is discussed below, with particular attention given to the specific environmental context.

### 5.1 Interplay between CTCs and neutrophilic cells

Neutrophils are the most abundant leukocytes in the circulation. They are considered to be “spectators” in the pathogenesis of cancer. Nevertheless, neutrophils comprise a heterogeneous population with high-density neutrophils (HDNs), low-density neutrophils (LDNs), normal-density neutrophils (NDNs), polymorphonuclear-myeloid-derived suppressor cells (PMN-MDSCs), and tumor-associated neutrophils (TANs) (Sagiv et al., 2015). A subpopulation of neutrophils takes on a major function in cancer progression, especially in metastasis (Kowanetz et al., 2010; Wculek and Malanchi, 2015; Hawila et al., 2017). The majority of WBCs detected in CTC-WBC clusters were characterized as neutrophils and, notably, patients with CTC neutrophil clusters had poorer progression-free survival rates compared to patients with pure single CTCs and CTC clusters. Inside a solid cancer, immune cells are capable of connecting with cancer cells to form tumor-immune hybrid cells (THCs) (Tanjak et al., 2024). It is hypothesized that THCs are a subgroup of cancer cells that are able to penetrate the bloodstream as circulating hybrid cells (CHCs) and form metastases. These results suggested a high predominance of CHCs and THCs in patients suffering from stage IV colorectal cancer (CRC). The THCs have been found to express CTLA4 in primary CRC lesions, showing a positive correlation with the upregulation of CD68, CD4, and HLA-DR levels within metastatic liver lesions, as seen in consensual molecular subtype (CMS) 1 of primary CRC tissue (Tanjak et al., 2024). Based on the pathway analysis of these genes, it seems THCs are linked to neutrophils because of an upregulation of neutrophil extracellular trap (NET) signaling routes and neutrophil breakdown cascades. These findings reveal molecular pathways involved in the generation of THCs, indicating fusion with neutrophils, which could promote cancer cell extravasation and subsequently metastasis. Moreover, the presence of cancer cells seems to support the intraluminal metastasis route. The fast interaction between CTCs and platelets in the blood (Labelle and Hynes, 2012) increases plasticity and the ability to start metastasis (Labelle et al., 2011) via processes such as RhoA–MYPT1–PP1-based YAP1 signal transduction (Haemmerle et al., 2017) and enhanced permeability of blood vessels through the interaction of ATP released from platelets with P2Y2 receptors (Russell and Monga, 2018; Gu et al., 2024). In cancer, TGF-β1 signal transduction can prevent tumor growth either by initiating apoptosis in premalignant cells or by interfering with the proliferation of cancer cells. Nonetheless, it can also enhance tumor growth through the initiation of EMT, activation of neoangiogenesis, and inhibition of anti-tumor T-cell reactions (Derynck et al., 2021). In addition, the activation of immunosuppressive TGF-β1 is stimulated in human cancers, but it is still uncertain which type of cells actually produce and liberate this protein. Therefore, the hypothesis was put forward that there are two sources of immunosuppressive TGF-β1. Nevertheless, the majority of cells produce latent TGF-β1 in conjunction with a partner protein that is covalently bound to LAP through two disulfide bridges. According to the cell type, the partner protein may be any one of four secreted latent TGF-β1 binding proteins (LTBP-1–4) or any one of two transmembrane proteins comprising leucine-rich repeats and commonly referred to as glycoprotein A repetitions predominant (GARP) and LRRC33. Production of GARP:TGF-β1 complexes leads to the surface display of latent TGF-β1 on GARP-expressing cells. GARP:TGF-β1 complexes have been reported on regulatory T cells (Tregs) stimulated via the T cell receptor (TCR), B cells stimulated by the B cell receptor, endothelial cells, mesenchymal cells, activated platelets, and megakaryocytes (Dedobbeleer et al., 2017; De Streel et al., 2020; Lecomte et al., 2023; Zhang et al., 2023). GARP-expressing Tregs (GARP + FOXP3+ cells) are a prominent cell type that produces TGF-β1, which suppresses T cell activity in human tumors. Glycoprotein A additionally shields against T cell-mediated clearances and natural killer (NK) cell-driven clearances and mainly involves GARP–TGF-β interaction (Schumacher et al., 2013) and the platelet-derived major histocompatibility complex class I (Rachidi et al., 2017). GARP interacts with the TGF-β signaling system via its ability to attach latent (inactive) TGF-β to the surface of cells such as Tregs and platelets and present it there (Van Meerbeeck et al., 2025). This kind of engagement is key to activating TGF-β, which then manages different biological processes such as immune tolerance and injury healing. This signaling pathway may also play a role in diseases such as cancer, where GARP can be utilized by cancer cells to promote evasion of the immune system (Van Meerbeeck et al., 2025). It has been suggested that, in human tumors, GARP-expressing Tregs constitute a source of TGF-β1 that suppresses neighboring tumor-infiltrating lymphocytes rather than blood endothelial cells (Van Meerbeeck et al., 2025).

To evaluate the metastatic capacity of CTC neutrophil clusters, individual CTCs, CTC clusters, and CTC neutrophil clusters that arose spontaneously in tumor-bearing mice were isolated. These were manipulated individually to inject an equal number of CTCs of each group into tail veins of receiving mice. Mice that were injected with CTCs from CTC neutrophil clusters metastasized considerably sooner compared to mice that were injected with pure CTCs (not connected with WBCs). Similarly, treatment of mice with palpable tumors using neutralizing antibodies directed against the neutrophil surface protein Ly-6G resulted in lower neutrophil infiltration at the primary site, total elimination of CTC neutrophil clusters, and a substantial decrease in metastasis, which resulted in increased survival. In contrast, mice that were injected with cancer cells and overexpressed G-CSF, which is a factor linked to the stimulation of neutrophils, displayed increased neutrophil infiltration into the primary tumor and a higher number of CTC neutrophil clusters in the vascular lumen, resulting in faster metastasis and reduced survival time. To gain an insight into the molecular characteristics underlying the elevated death rate of neutrophil-associated CTCs, a recently established protocol for parallel DNA and RNA sequencing of single cells has attracted considerable attention (Macaulay et al., 2016). Exome sequencing identified a number of genes, among them TLE1, that were mutated solely in patients with CTC neutrophil clusters. Primary solid tumors generated by cells that were modified to express specific TLE1 mutations exhibited enhanced neutrophil infiltration and released considerably more CTC neutrophil clusters into circulation. Single-cell RNA sequencing analysis of CTCs either with or without the presence of linked neutrophils revealed that CTCs of CTC-neutrophil clusters overexpress positive modulators of cell cycle and DNA replication, implying that CTCs gain a proliferative benefit from engagement with neutrophils. Analysis of the simultaneous expression of cytokines and their receptors within CTCs and neutrophils from heterotypic clusters indicated that IL-6 and IL-1 signaling pathways are important elements of the CTC-neutrophil interaction, which results in enhanced multiplication of cancer cells. Most interestingly, the majority of CTCs found in CTC-neutrophil clusters expressed G-CSF and other cytokines implicated in stimulating neutrophils, which probably encourages a positive feedback circuit that reinforces the proliferative ability of the cancer cells. All of this indicates that neutrophils promote the intraluminal route of cancer metastasis.

**FIGURE 12:**
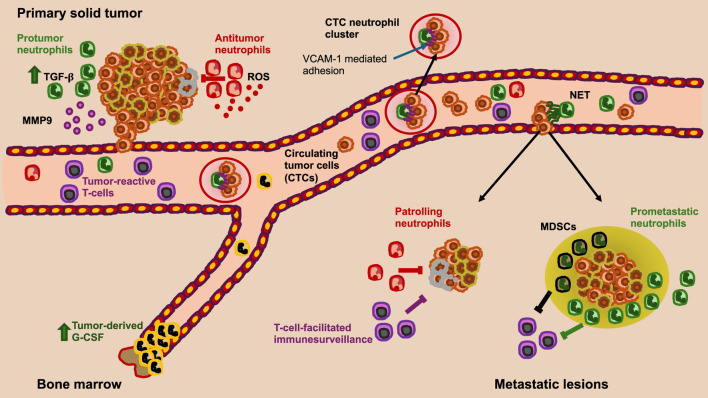
Illustrating scheme of how neutrophils act in the various steps of the metastatic cascade. Infiltrating neutrophils within the primary solid tumor can exert either antitumor/cytotoxic effects or pro-tumor functions, such as angiogenesis and intravasation, which are triggered by MMP9. The interplay of cancer cells and neutrophils, which is set up by VCAM-1, facilitates the intravasation of CTC-neutrophil clusters, which possess a higher capacity for metastasis compared to CTCs that are not interacting with neutrophils. Neutrophils can also stimulate extravasation by creating neutrophil extracellular traps (NETs). Finally, protumoral neutrophils suppress cytotoxic T lymphocytes at the metastatic site, exerting an effect analogous to that of myeloid-derived suppressor cells (MDSCs), while patrolling neutrophils, in cooperation with antitumoral T cells, limit metastatic colonization.

Since targeted elimination of all neutrophils could have undesirable adverse effects, an investigation was conducted to explore options for specifically combating CTC-neutrophil clusters. They developed a CRISPR-Cas9 -based mini-pool screening system for loss-of-function analysis *in vivo*, which targets cell-cell junction pairs that are expressed simultaneously in CTCs and neutrophils originating from heterotypic clusters and revealed that abrogating VCAM1 expression in CTCs abolished their capability to attach to neutrophils (Figure 12). It is well established that an elevated neutrophil count frequently coincides with poor treatment outcomes, but it remains unclear whether and how tumor-associated neutrophils are involved in tumorigenesis. Neutrophils can directly facilitate cancer progression through various mechanisms, from enhancing angiogenesis and suppressing the cytotoxic T-cell activity to generating NETs that favor metastasis, as discussed in Powell and Huttenlocher (2016). Utilizing intravital microscopy, Cools-Lartigue and coworkers demonstrated that neutrophil-generated NETs foster CTC attachment to capillaries and subsequently cancer cell extravasation into target organs (Cools-Lartigue et al., 2013). Another article demonstrated that neutrophils residing in CTC clusters also facilitate tumor growth by supporting CTC proliferation (Aceto et al., 2014), both while circulating and after reaching distant organs (Szczerba et al., 2019). Nevertheless, it was hypothesized that neutrophils interfere with tumor growth by enhancing the removal of tumors and stimulating the adaptive immune defense. It is assumed that contextual polarization into antitumor (N1) or protumor (N2) neutrophils may account for this obvious discrepancy (Fridlender et al., 2009; Powell and Huttenlocher, 2016).

### 5.2 Missense mutation in TLE1 of neutrophils leads to their enlisting toward the primary tumor

The mechanism by which missense mutations in TLE1 result in the recruitment of neutrophils toward the primary tumor and amplify the generation of CTC neutrophil clusters is still unclear. It is appealing to hypothesize that the elevated G-CSF levels seen in CTCs from mice with heterotypic clusters are related to the activation of NF κ B activity that may be caused by the TLE1 mutation (Ramasamy et al., 2016). In addition, elevated G-CSF concentrations may promote the differentiation of neutrophils into a protumoral phenotype (Pylaeva et al., 2016). Cancer cells injected intravenously into naive mice, either depleted or not depleted of neutrophils, exhibited no variation in metastatic growth, indicating that prior cancer cell-neutrophil interactions at the primary tumor site are critical for neutrophils to facilitate disease advancement. This is substantiated by a trial which has demonstrated that while reducing neutrophil counts during primary tumor proliferation decreases metastasis, reducing neutrophil counts after resection of the primary solid tumor does not (Coffelt et al., 2015). Another outstanding question is what effect other kinds of white blood cells attached to CTC clusters have on tumor propagation. In the blood of patients, 25% of CTC-WBC clusters are associated with lymphocytes; however, the implications of this relationship still need to be investigated. In conclusion, the work of Szczerba and coworkers (Szczerba et al., 2019) reveals a mechanism whereby cancer cells benefit from their engagement with neutrophils during their spread to enhance their metastatic potential. These insights raise the prospect of addressing the regulatory drivers of CTC-neutrophil cluster generation to mitigate the metastatic capacity of cancer cells. Nevertheless, it can be proposed that the blockage of neutrophil-metastatic cancer cell interactions may induce the extraluminal route of cancer metastasis, in which the spreading cancer cells do not need to be protected from the immune system (Figure 12).

### 5.3 Can neutrophilic cells promote the new alternative route of metastasis?

Metastatic cancer cells secrete large amounts of chemokines and proteins to recruit immune cells and promote angiogenesis, thereby fostering the emergence of a singular inflammatory and highly vascularized cavity. The proposed neutrophil subpopulation that supports the pathological progression of cancers like initiation, invasion, and metastasis primarily consists of LDNs, comprising both the mature subtype of LDNs and the immature low-density PMN-MDSCs (Wright et al., 2017; Rahman et al., 2019). Fridlender and colleagues characterized a duality in TANs that were identified inside and surrounding solid tumors (Fridlender and Albelda, 2012; Shaul and Fridlender, 2019). Due to their heterogeneity and plasticity, neutrophils can have either a tumor-inhibiting or tumor-promoting effect. The anti-tumor and pro-tumor neutrophils were termed N1 and N2 by Fridlender and collaborators (Fridlender et al., 2009). In mice, they proved that neutrophils that reach the vicinity of the primary tumor can evolve into either antitumor N1 cells or pro-tumor N2 cells. The choice between the two appears to rely on the secretion of TGF-β in the TME, which enhances pro-tumorigenic N2 cells (Fan, 1999; Fridlender et al., 2009). This functional differentiation is triggered by local tissue/tumor cues and can be regarded as plasticity rather than a true subtype (Koenderman and Vrisekoop, 2024). N1 anti-tumor activity can be governed in many ways. For instance, the killing of cancer cells induced by neutrophils relies on the release of nitric oxide triggered by hepatocyte growth factor (HGF)/MET (Finisguerra et al., 2015). N1 neutrophils may also prevent metastasis in the lungs by producing hydrogen peroxide (H_2_O_2_) that is facilitated through chemokine ligand 2 (CCL2) released from the tumor (Granot et al., 2011). Additional mechanisms implicated in the antitumor activity of neutrophils comprise the stimulation of cancer cell apoptosis through direct engagement or tumor necrosis factor-related apoptosis-inducing ligand (TRAIL) release, antibody-dependent cellular cytotoxicity (ADCC), and the activation of T-cell functioning (Fabian et al., 1987). Conversely, N2 neutrophils stimulate the growth of tumors and metastasis, as will be discussed in more detail below.

The function of neutrophils in supporting (e.g., N2-type via NETs and CTC clusters) or suppressing (N1-type) cancer metastasis is based on the context. The context itself can be a decisive factor affecting the decision of cancer cells between at least two signaling pathways: “exposed to the immune system” (intraluminal metastasis route) or “withdrawn from the immune system” (extraluminal metastasis route). It is a two-sided phenomenon that serves as a crucial mediating factor between cancer cells and the immune system. Neutrophils are either involved in promoting metastasis (pro-tumorigenic, N2-type) or preventing it (anti-tumorigenic, N1-type), depending on signals such as mechanical cues, including stiffness and viscoelasticity from the TME. An immune surveillance mechanism of cancer cells when penetrating the lumen of blood vessels is the formation of CTC clusters. CTC clusters, especially in combination with N2-type neutrophils and NETs, constitute a highly metastatic, pre-tumorigenic, and chemoresistant entity circulating in the bloodstream. These heterotypic clusters can develop metastases 23 to 50 times more effectively than individual CTCs (Aceto et al., 2014). These neutrophils adhere to CTCs, frequently in conjunction with platelets, forming heterotypic clusters. Inside these clusters, neutrophils release DNA and build NETs. These NETs are a kind of trapping mechanism for CTCs, offering a protective and encouraging microenvironment (pre-metastatic niche) that improves survival, shields against fluid shear stress, and supports invasion (Obeagu, 2026). Inside NETs, activated neutrophils can liberate DNA networks and proteases that entrap CTCs, promote their adhesion to capillaries, and facilitate metastasis to distant organs, notably the lungs. These CTC-neutrophil/platelet clusters in the bloodstream offer survival benefits, shielding them from immune surveillance (death caused by natural killer cells (NK cells)) and consequently offer immune evasion. Moreover, the CTC-neutrophil/platelet clusters enhance their proliferation. At the molecular level, pro-tumor N2-type neutrophils release TGF- β , MMP-9, and IL-17, which promote an immunosuppressive TME, increase angiogenesis, and favor epithelial-mesenchymal transition (EMT) to elevate metastatic capacity. In addition, N2-type neutrophils in these clusters secrete factors, including TNF-α, OSM, IL-1β, and IL-6, which encourage the proliferation of cancer cells. CTC clusters have a better survival rate in the bloodstream because they have a higher degree of stem cell similarity due to their hypomethylation of transcription factor binding sites for OCT4, NANOG, and SOX2, enabling them to bind DNA. In summary, N2-type neutrophils promote the survival and metastasis of CTC clusters by producing NETs, thereby establishing a specialized, highly aggressive micro-niche in the hematologic circulation. In contrast, anti-tumorigenic N1-type neutrophils exert a cytotoxic role and enable immune surveillance. N1-type neutrophils, which are frequently marked as having a high-density or activated phenotype, can directly destroy cancer cells by secreting ROS and H_2_O_2_. Moreover, they can support immune surveillance and suppress metastasis through the release of factors such as TNF*α* and the activation of antibody-dependent cellular cytotoxicity (ADCC).

There is a context-dependent influence on the polarization of neutrophils. Neutrophil polarization (N1-type versus N2-type) is controlled by the TME and determines whether cancer cells take an exposure route, which is susceptible to the immune system and is frequently N1-type dominated, or a concealment route, which escapes the immune system and is frequently N2/NET-type dominated. In intracellular/hidden mode, pro-tumor neutrophils (N2/NETs) establish a protective, typically immunosuppressive milieu that supports CTC survival and enables them to avoid detection by the immune system, for example, attack by NK cells. In exposed mode (immune-oriented mode), anti-tumor neutrophils (N1) are actively involved in killing antibody-opsonized cancer cells. This dynamic plasticity enables cancers to influence neutrophils, frequently leading to a transition to a tumor-promotive N2 phenotype in later stages of metastasis, as demonstrated in the “piggyback” (back-to-back) interaction in CTC clusters.

Similar to macrophages (Zhou et al., 2015), N1 and N2 neutrophils can transition into one another according to specific tumor-derived determinants. TGF-β is an extensively examined molecule that acts as a mediator in the polarization of neutrophils to the N2 phenotype (Fridlender et al., 2009). Both TGF-β inhibition and depletion of neutrophils can stimulate the N1 phenotype and significantly reduce the growth of tumors in mouse models. Interleukin-35 (IL-35) (Zou et al., 2017) fosters the generation of granulocyte-colony stimulating factor (G-CSF) and IL-6, activates signal transducer and activator of transcription 3 (STAT3) and extracellular signal-regulated kinase (ERK) signal transduction routes in neutrophils, and enhances the expression of inducible nitric oxide synthase (iNOS) to subsequently prevent T cell activity. Moreover, studies have demonstrated that type I interferon (IFN) can help convert neutrophils into the N1 anti-tumor phenotype (Andzinski et al., 2016; Siakaeva et al., 2019). In the paucity of IFN-β, NET expression is low within primary lesions and the pre-migratory lung, whereas the application of interferon therapy in mouse tumor models leads to the polarization of neutrophils toward the N1 anti-tumor phenotype (Andzinski et al., 2016). Cytokine concentration and alterations in the TME, such as hypoxia, may also be relevant for the polarization of neutrophils. Neutrophils have been shown to exhibit an activated phenotype with antitumor effects to stimulate T-cell reactions in the initial tumor phases (Eruslanov et al., 2014). In contrast, the primary solid tumor progressively develops into the N2 phenotype of neutrophils. In addition, N1 neutrophils live for a shorter period of time, although they exhibit greater maturity and cytotoxicity compared to N2 neutrophils. Hence, converting neutrophils from the N2 to N1 phenotype could restore the antitumor immune responsiveness of neutrophils. Another neutrophil phenotype that displays relatively high PD-L1 expression was seen in cancer patients (Xue et al., 2022). This subset exhibited an IFN-triggered transcription pattern in an acute inflammation model. In fact, high levels of IFN-γ *in vitro* were able to stimulate the expression of PD-L1 on neutrophils from healthy control subjects (De Kleijn et al., 2013), further demonstrating that the cells block the cytotoxicity of T cells (Koenderman and Vrisekoop, 2024).

## 6 How are organ-specific tropism mechanisms impacted by the route of metastasis?

Organ-specific tropism in metastases refers to the propensity of cancer cells to disseminate and settle preferentially in certain distant organs rather than spreading randomly across the entire body. The choice of the route of metastasis has a significant influence on organ-specific tropism by affecting the premetastatic niche, which is a prematurely established, organ-specific environment that is essential for colonization. The route, whether it is via the bloodstream or the lymphatic system, defines the very first interactions with the microenvironment, including interferences with organ-specific endothelial cells and infiltration of specific immune cells. These reciprocal actions shape the organ-specific immune scene and its metabolic microenvironment, ultimately affecting which molecular programs are needed for CTCs to survive, adjust, and multiply at the remote site. In the bloodstream route, cancer cells that penetrate the bloodstream encounter certain kinds of challenges, such as immunological surveillance and mechanical stress. Successful colonization relies on the capacity to survive in the bloodstream, evade detection by the immune system, and extravasate across the capillary beds to distant organs. The specific characteristics of an organ, such as its vascular system, determine which cells are able to successfully attach and escape. For instance, the liver is a prevalent site because of its richness of vascular supply, but its successful colonization demands conquering specific metabolic and immunological hurdles of the hepatic microenvironment. The lymphatic route, or lymphangiogenesis, can result in metastases in local lymph nodes and, possibly, in organs further away. The route of lymphatic dissemination, which is determined by parameters such as tumor-expressed growth factors and the existence of lymphatic vessels, decides what secondary locations are most prominent to be populated.

The primary tumor determines which organ will be the site of the new pre-metastatic niche. First of all, each organ comprises its own specific microenvironment, referred to as the organ-specific microenvironment. The organ provides a “soil” that is either susceptible or adverse to the cancer cell, which is referred to as “the seed”. The specific microenvironment of the organ encompasses its individual (unique) immune cells, stromal constituents, and metabolic circumstances that cancer cells have to accommodate to. Another prerequisite for the successful development of secondary tumors is the development of a pre-metastatic niche. Prior to the arrival of cancer cells, the primary tumor may release regulatory factors, like extracellular vesicles, to precondition the targeted remote organ. These pre-metastatic niches are able to act as suppressors of the local immune response, stimulate angiogenesis, and restructure the ECM scaffold, thereby rendering it more favorable for the entry and growth of cancer cells. The pathway of metastasis, such as blood or lymph, influences which organ-specific pre-metastatic niche is formed, and these can vary considerably. The extraluminal route of cancer cell metastasis could play an even greater role here, as the cancer cells arrive at the target organ in a less weakened state and can adapt gradually as they travel through the tissue. In this way, cancer cells could also gather immune cells around them that can optimally support them in establishing the pre-metastatic niche. This non-random behavior of metastasis is a crucial feature of cancer biology and has significant implications for disease progression, as cancer cells often adapt at multiple levels: cellular, molecular, and genetic. In terms of metabolic and molecular adjustments, cancer cells have to modify their metabolism to the requirements of the new organ to obtain energy and nutrients. This involves adapting to specific metabolites and circumstances such as hypoxia, which may be specific to certain organs. These adjustments can be decisive for their survival in the face of oxidative stress and a lack of nutrients. Genetic and cellular adjustments involve specific genes and cellular behavior that are much more appropriate for specific organ settings. For example, certain lung-tropic clones display distinct post-transcriptional regulatory mechanisms, whereas other genes may favor tropism for the brain or liver. The metastatic route chooses the specific molecular and cellular processes that are most appropriate for the targeted organ. A more precise description is provided in the following. What does organ-specific tropism mean exactly in cancer? Metastasis is not random: instead of spreading to any organ, cancer cells prefer certain organs for metastasis. For example, prostate cancer frequently metastasizes to the patient’s bones, whereas colon cancer often disseminates to the liver and lungs. How does this impact the patient’s prognosis? This organ-specific response is an important determinant of cancer prognosis and treatment recommendations. Why does organ-specific metastasis occur? Several factors actually cause organ-specific tropism. There are tumor-intrinsic factors. For instance, cancer cells exhibit characteristics that enable them to multiply more readily in specific organs. Moreover, the particular microenvironment of each organ, comprising the availability of growth factors, immune cells, and other constituents, may either encourage or hinder the growth of cancer cells. Another factor is the generation of the pre-metastatic niche. Tumors are able to precondition a specific organ for metastasis in that they establish a premetastatic niche before the arrival of cancer cells, thereby rendering it more susceptible to metastatic colonization. Another factor contributing to organ-specific metastasis is the interaction of cancer cells with the ECM. For example, cancer cells can directly engage with the ECM of various organs, which can impact their capacity for invasion, migration, and survival within a novel, challenging microenvironment. The characteristics of the circulatory system represent another factor that appears to play a decisive role. The manner in which cancer cells migrate through the circulatory system can determine which organs they are predisposed to access. Finally, there are metabolic adaptations. Cancer cells can adjust metabolically to the specific requirements of various organs, which can have an impact on their survival and proliferation.

What is the implication of cancer treatment? There is one approach in which the targeting of specific organs is the focus. Consequently, a deeper understanding of organotropism can assist scientists in designing therapies that address the specific mechanisms driving metastasis to certain organs. Another approach involves the impairment of the pre-metastatic niche generation. For instance, preventing the creation of premetastatic niches could stop cancer cells from settling in remote organs. The modulation of the microenvironment, such as the TME, appears to be a promising approach. Therefore, altering the host’s microenvironment to render it less host-friendly to cancer cells is an additional possible therapeutic approach. After all, organ-specific tropism is complicated and is instrumental in metastasis. By improving the comprehension of the factors that fuel this process, scientists can design more powerful cancer metastasis prevention and treatment strategies.

## 7 Discussion, conclusion, and future directions

In conclusion, three routes of metastasis have been identified: the first, the intraluminal route of metastasis, is universal and has been studied most frequently. It also forms the basis for the globally accepted and dominant Hallmarks of Cancer definitions. The second, the new extraluminal metastasis route, has not yet been investigated as extensively. The results are promising, and it appears to be equally universal in nature, as it is found in an increasing number of cancer types. The third, the interfacial metastasis route, has been the least thoroughly studied of the three. This third route has been discussed in detail in this review.

These three metastasis pathways can each be used as a continuous metastasis pathway by malignant cancer cells of a specific cancer type. In this case, malignant cancer cells could all choose a single, specific metastasis route, exhibiting a uniform metastasis profile. This uniform pattern of metastasis is not the only one possible. Rather, there are different approaches that malignant cancer cells adopt to metastasize. For instance, the three routes of metastasis can be used either exclusively or alternately by cancer cells. It is even possible that there is a heterogeneous distribution of the three metastasis routes within a single tumor. The nature of the alternating mode is not yet known, but it appears that the mechanical characteristics of the ECM environment, such as stiffness and viscoelasticity, may have an influence. The selection of guiding principles for mechanical properties is an important hypothesis that requires experimental verification. Specifically, it can be proposed that experimental models, such as 3D matrices with controllable stiffness and viscoelasticity, can be used, wherein endothelial vessels can be formed for malignant cancer cells to migrate along their metastatic pathway, either intraluminally, extraluminally, or interfacially. Their migration and invasion route can be monitored using 3D confocal imaging. Alternatively, advanced *in vitro* platforms, such as microfluidic chips, also known as a “vessel-on-a-chip”, can be utilized to simulate the physical, mechanical, cellular, and biochemical environment of human blood vessels. This platform can generate precise fluid flow dynamics via controlled physiological shear stress, to which intraluminal metastatic cancer cells are then exposed. The microfluidic chips can simulate certain microenvironments, like tumor-associated vascular networks with disrupted vascular glycocalyx, to explore how hemodynamics and vascular geometry impact cancer cell adhesion and subsequently the selection of the metastasis route. The viscoelastic properties of the TME function as a powerful external regulatory agent, forcing cancer cells to alter their internal mechanical characteristics (viscoelasticity), which in turn encourages a more aggressive, malignant, and migratory cancer phenotype. The elevated stiffness and modified viscoelasticity of the TME can suppress the functionality of immune cells, like T cells, and encourage the differentiation of regulatory T cells (Tregs), resulting in immune evasion by cancer cells. In addition, *in vivo* imaging in mouse models can be analyzed for the metastasis of cancer cells. All of these experimental approaches can be used to test hypotheses. These model systems could also be used to test certain drugs that alter the stiffness or viscoelasticity of the ECM scaffold, e.g., the hydrogel.

In summary, the following important findings can be noted: first, the stiffness and viscoelastic properties of the ECM could influence the choice of metastasis pathway. Second, the extraluminal metastasis pathway is universal in nature, although the intravascular route has been identified in almost all cancer types. Third, cancer cells from a primary tumor can choose between three metastasis pathways, whereby the selection of a specific metastasis pathway is always uniform within a given cancer or cancer type or the selection of the metastasis pathway can alternate between the three pathways, resulting in a heterogeneous distribution of metastasis pathways within a specific cancer type. Fourth, cancer cells may switch between metastasis routes depending on TME signals. Moreover, it can be concluded that these two new paradigms for the choice of a metastasis route may significantly hinder the development of new effective anti-metastatic therapies. Therefore, for example, targeted manipulation of embryonic factors associated with migration and identified in cancer metastasis could be particularly important for pericytic migration/extravascular migratory metastasis. Fifth, the extraluminal metastasis route appears to be a universal phenomenon, as it has been reported in several different cancer types. However, the majority of findings continued to be detected in melanomas and gliomas. Sixth, both the intraluminal and extraluminal metastasis routes are connected to EMT and/or hybrid-EMT variations. It appears likely that the interfacial metastasis route is also linked to EMT and/or hybrid-EMT variations. It can be hypothesized that they are also connected to jamming-to-unjamming transition. Mechanobiological aspects are expected to play a significant role and will be systematically analyzed across different cancer types. Specifically, tests will be conducted to determine whether softer cells may be better suited for intraluminal pathways to pass through the vessel lining and withstand the shear force of blood flow or stiffer cancer cells that may be better suited for extraluminal pathways to adhere to the basement membrane. This hypothesis assumes that the mechanical characteristics of cancer cells do not change or adapt dynamically as a result of intravasation or 3D migration through the TME, which may not correspond to real-life conditions, as the forces exerted (as indicated by fiber displacements) by invasive human breast cancer cells on a 3D collagen matrix fluctuate significantly over time (Hayn et al., 2023). Moreover, these matrix displacements are dependent on matrix composition (e.g., fibronectin cross-linking collagen fibers) and, thereby, on matrix stiffness (Hayn et al., 2023).

A variety of mechanobiological methodologies have been developed to measure mechanical forces inside the TME, including *in vivo*, *in vitro*, and patient models (Zhang et al., 2025). These techniques encompass 3D traction force microscopy, molecular force sensors (DNA-or fluorescent protein-based), ultrasound elastography (real-time imaging of stiffness), magnetic resonance elastography, and optical coherence tomography elastography with micron-based resolution (Xin et al., 2023). Apart from measuring the mechanical properties of the environment, the mechanical cues of the TME can be altered by exerting tugging forces on cancer cells. Moreover, there are biophysical approaches available that can exert forces on cancer cells. For instance, tugging forces exerted via magnetic beads decrease β3 expression and increase cofilin activity to promote the invasion of fibrosarcoma cells (Gasparski et al., 2017). The stiffened cancer tissue triggers integrin activation and subsequent signaling processes, which frequently imparts a more malignant phenotype to cancer cells (Paszek et al., 2005; Artym et al., 2015). Blocking collagen cross-linking caused by lysyl oxidase (LOX) reduces tissue fibrosis and stiffness, which lowers the incidence of cancer through β1 integrin-PI3K signal transduction (Levental et al., 2009). Increased tissue stiffness results in a voluminous glycocalyx on the plasma membrane, which controls integrin clustering and the interaction between integrins and the ECM scaffold to activate the FAK and ERK signaling cascades that promote the survival and growth of breast and brain cancer cells (Paszek et al., 2014; Barnes et al., 2018). Integrin-facilitated mechanotransduction has a reciprocal effect on the mechanical properties of the TME. It occurs in the following way: solid cancers are physically stiff, mostly because of too high collagen deposits and CAFs. Consequently, this results in mechanical stress on the cancer cells. How does the stiffness of the surrounding environment impact cancer cells? A dense ECM elevates stiffness, which cancer cells perceive via surface receptors such as integrins. The integration of these mechanical signals prompts the cells to proliferate and migrate. What effect does solid stress have on cancer cells? Fast growth leads to physical compression. Contrary to general expectations, this pressure can increase the invasiveness of cancer cells. What influence does fluid pressure have on cancer cells and cancer treatment? High interstitial fluid pressure hinders the delivery of active drugs and encourages metastasis through the activation of signaling pathways that support migration.

Finally, the review will call into question the traditional notion of concentrating solely on intravascular spreading and suggest that the mechanical microenvironment functions as the “decision maker” of the metastatic route. These findings will have a considerable influence on future basic research and the development of treatment methods. For instance, in addition to investigating the mechanical characteristics of the TME, it may be proposed to design combined treatment approaches that target both transfer pathways or to identify biomarkers that differentiate between cancer types based on different transfer pathways. In the future, research approaches should investigate the effect of the mechanical properties of the TME, such as stiffness (ECM remodeling and collagen density) and viscoelasticity (fluid flow and stress relaxation), on the choice of metastasis route. In addition, the mechanical properties of cancer cells and the effect of interacting cells, such as immune cells, for example, neutrophils and/or endothelial cells, should also be explored over time. Mechanobiological aspects are expected to play a significant role and will be systematically analyzed for the different cancer types. In conclusion, it can be said that the hallmark of cancer, “inducing or accessing the vasculature”, should be redefined in more general terms as “interaction with the endothelium” due to the two alternative routes of intraluminal metastasis of cancer cells.
